# MicroRNAs from saliva of anopheline mosquitoes mimic human endogenous miRNAs and may contribute to vector-host-pathogen interactions

**DOI:** 10.1038/s41598-019-39880-1

**Published:** 2019-02-27

**Authors:** Bruno Arcà, Alessio Colantoni, Carmine Fiorillo, Francesco Severini, Vladimir Benes, Marco Di Luca, Raffaele A. Calogero, Fabrizio Lombardo

**Affiliations:** 1 Department of Public Health and Infectious Diseases, “Sapienza” University, Piazzale Aldo Moro 5, 00185 Rome, Italy; 2 Department of Biology and Biotechnology, “Sapienza University”, Piazzale Aldo Moro 5, 00185 Rome, Italy; 3 Department of Infectious Diseases, Istituto Superiore di Sanità, Viale Regina Elena 299, 00161 Rome, Italy; 4 Genomics Core Facility, European Molecular Biology Laboratory, Meyerhofstrasse 1, 69117 Heidelberg, Germany; 5 Department of Molecular Biotechnology and Health Sciences, University of Turin, Via Nizza 52, 10126 Turin, Italy

## Abstract

During blood feeding haematophagous arthropods inject into their hosts a cocktail of salivary proteins whose main role is to counteract host haemostasis, inflammation and immunity. However, animal body fluids are known to also carry miRNAs. To get insights into saliva and salivary gland miRNA repertoires of the African malaria vector *Anopheles coluzzii* we used small RNA-Seq and identified 214 miRNAs, including tissue-enriched, sex-biased and putative novel anopheline miRNAs. Noteworthy, miRNAs were asymmetrically distributed between saliva and salivary glands, suggesting that selected miRNAs may be preferentially directed toward mosquito saliva. The evolutionary conservation of a subset of saliva miRNAs in *Anopheles* and *Aedes* mosquitoes, and in the tick *Ixodes ricinus*, supports the idea of a non-random occurrence pointing to their possible physiological role in blood feeding by arthropods. Strikingly, eleven of the most abundant *An. coluzzi* saliva miRNAs mimicked human miRNAs. Prediction analysis and search for experimentally validated targets indicated that miRNAs from *An. coluzzii* saliva may act on host mRNAs involved in immune and inflammatory responses. Overall, this study raises the intriguing hypothesis that miRNAs injected into vertebrates with vector saliva may contribute to host manipulation with possible implication for vector-host interaction and pathogen transmission.

## Introduction

Mosquitoes are vectors of parasitic and arboviral diseases of great importance to human health. Malaria, which is transmitted by *Anopheles* mosquitoes, affected >200 million people with ~450 thousands deaths in 2016^1^ and dengue, transmitted by *Aedes* mosquitoes, may be responsible for >100 million symptomatic infections per year^2^. Most vector-borne pathogens, as malaria parasites and dengue viruses, are transmitted to vertebrates through hematophagous arthropod saliva during the blood meal. Saliva of blood feeding arthropods (BFA) is a complex cocktail including hundreds of salivary proteins and its role in hematophagy is pretty well known^3–6^. As far as mosquitoes are concerned, transcriptomic^4,7–15^, genomic^16–18^ and proteomic studies^19–22^ allowed to clarify that mosquito saliva carries ~100–150 salivary proteins whose main role is to facilitate blood feeding by counterbalancing host responses to tissue injury, namely haemostasis, inflammation and immunity^23^. Moreover, in virtue of its immunomodulatory properties, mosquito saliva generates at the biting site a local environment that may affect pathogen transmission^24–30^.

MicroRNAs (miRNAs) are small non-coding RNAs of ~22 nt in length with a relevant role in post-transcriptional gene regulation. Typically, primary miRNA transcripts (pri-miRNAs) are first processed to hairpins of ~80 nt in length (pre-miRNAs) and then into the mature miRNA duplex^31,32^. One strand of the duplex, named the guide strand, is preferentially loaded into the miRNA-induced silencing complex (miRISC) and drives it to the target mRNA promoting its degradation or translational inhibition^31,33,34^. Target recognition mainly involves imperfect base pairing between the mRNA 3′UTR and the miRNA, with the seed region of the miRNA (nucleotides 2 to 8) playing a crucial role in target selection^31,33,35^. miRNAs are essentially found in all animal cell types where they exhibit tissue-specific expression patterns and, as part of complex networks, contribute to the regulation of practically every aspect of cell life, from cell growth and differentiation to apoptosis, development and immunity^31,36^. miRNAs are not only present within cells but also extracellularly. They have been found in all human body fluids^37–39^, as well as in the saliva of disease vectors as *Aedes* mosquitoes and *Ixodes* ticks^40,41^. Extracellular miRNAs in body fluids may be either in complex with proteins, as Argonaute (Ago) family members or High Density Lipoproteins, or may be carried within exosomal microvesicles^42–44^. The role of extracellular miRNAs is still debated^44,45^ but there is clear evidence that miRNAs enclosed within exosomes may play roles in cell-cell communication^46–48^. In this scenario miRNAs carried by exosomes may enter the target cells by direct fusion to plasma membranes or receptor-mediated endocytosis, whereas the vesicle-free miRNAs bound to Ago proteins may find their way through gap junction channels or some other yet unknown mechanism^45,49^.

miRNAs typically target endogenous genes, nevertheless, it is known that viral-encoded miRNAs target host mRNAs within infected cells^50^. In addition, exosomal miRNAs from parasitic nematodes may target host genes associated with immunity and inflammation^51,52^. Overall, these observations raise the fascinating hypothesis that miRNAs in mosquito saliva, perhaps encapsulated within exosomes, are injected into vertebrate hosts during blood feeding and may represent additional players in vector-pathogen-host interactions, contributing to manipulation of host inflammatory and immune responses.

Anopheline miRNAs have been studied in different experimental conditions in the malaria vectors *Anopheles gambiae*^53–56^, *An. coluzzii*^57,58^, *An. stephensi*^59–62^, *An. funestus*^63^, *An. anthropophagus*^64^ and *An. sinensis*^65,66^. However, so far no information has been obtained on the presence of miRNAs in the saliva of *Anopheles* species. To get insights into anopheline saliva miRNA composition and verify whether saliva-enriched miRNAs may have the capacity to manipulate host responses, we carried out a small RNA-Seq study on adult female salivary glands and saliva of the African malaria vector *An. coluzzii* using adult males and females as reference.

## Results

### Deep sequencing of small RNAs from *Anopheles coluzzii*

*Anopheles coluzzii* saliva (S), salivary glands (G), adult males (M) and females (F) were collected as described in the method section. Samples were in triplicate; for the saliva sample a pilot study including a duplicate was previously performed and, therefore, a total of five saliva replicates were analysed in this study (Supplementary Fig. S1A). Overall, 14 small RNA libraries were constructed and used for Illumina high-throughput sequencing, yielding a total of ~180 million reads (S = 38.80, G = 42.85, F = 48.65, M = 49.57). After quality filtering, adapter trimming and size selection (≥14 nt) approximately 126 million reads were retained for mapping to the *An. gambiae* genome (PEST strain, AgamP4 assembly), a choice motivated by its more complete assembly and annotation and the very close evolutionary relationships between *An. gambiae* and *An. coluzzii* (see methods). Approximately 84 million reads aligned to AgamP4 (S = 7.75; G = 21.06; F = 28.77; M = 26.46) and were mapped to rRNAs and to a list of 273 miRNA precursors (Supplementary File S1) and other ncRNAs (Table 1). Counts mapping to hairpins and other ncRNAs were used to investigate the linear relationships between replicates of the different samples. The correlation was very high for G, F and M (Pearson’s coefficients in the range 0.88–0.99) and moderate to high for the five S replicates (0.41–0.92, Supplementary Fig. S1B). Variation between libraries was also evaluated calculating distances based on fold change and biological coefficient of variation. Samples G, F and M formed three distinct clusters with little variation among replicates, whereas the five saliva libraries appeared more heterogeneous, but still clustering together and independently from the other samples (Supplementary Fig. S1C).

**Table 1:** Summary of deep sequencing of small RNAs from *An. coluzzii*.

sample	raw reads	filtered reads	AgamP4	rRNAs	ncRNAs
S	38.807	27.875	7.758	5.295	0.193
G	42.856	27.458	21.069	13.262	1.880
F	48.652	36.762	28.771	9.023	6.756
M	49.570	33.875	26.466	11.854	8.729
total	179.885	125.970	84.065	39.436	17.559

Numbers indicate million reads. S, saliva; G, female salivary glands; F, adult females; M, adult males. Filtered reads, reads remaining after adapter removal and size selection (≥14). Reads mapping to the *An. gambiae* genome (AgamP4), ribosomal RNAs (rRNAs) and to a list of 273 miRNA precursors plus other non-coding RNAs (ncRNAs) are indicated.

### Size distribution and mapping

**Figure 1:**
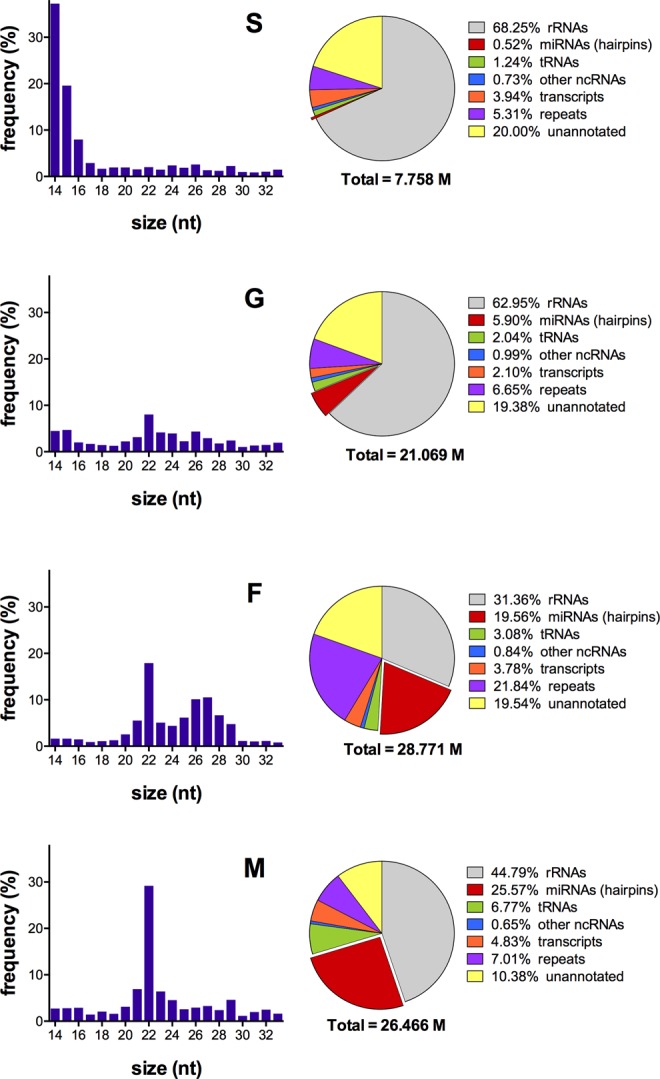
Features of small RNA sequenced from the four *Anopheles coluzzii* samples. The bar plots on the left show the frequency and size distribution of reads 14–33 nt in length mapping to the *An. gambiae* genome (AgamP4) and subtracted of those mapping to rRNAs. The pie charts on the right summarize the results of read alignment to rRNAs, miRNA precursors, tRNAs, other non-coding RNAs (including snoRNAs, snRNAs, Metazoa SRP, arthropod 7SK and RNase P) and to *An. gambiae* transcripts and repeats. Unannotated, reads mapping to AgamP4 but no to the other classes. Total, number of reads mapping to AgamP4. S, saliva; G, salivary glands; F, adult females; M, adult males.

Reads mapping to the *An. gambiae* genome, excluding those representing ribosomal RNAs, were analysed for their size distribution. The G, F and M samples showed a peak at 22 nt, which is typical of mature miRNAs and was prominent in the M sample, less pronounced in F and smaller in the G sample (Fig. 1, left panels). The F sample also showed a secondary peak in the range 25–29 nt likely representing piRNAs; a similar pattern was reported in a previous study on *An. gambiae* ovaries^54^. No peak of ~22 nt was visible in the S sample, with most of the reads falling in the 14–16 nt range, which is indicative of degradation that most likely occurred because of the elaborated saliva collection procedure. Nevertheless, ~0.23 million reads from the S sample mapped in the 20–24 nt range.

The result of reads alignment to rRNAs, miRNA precursors, tRNAs, other non-coding RNAs and to *An. gambiae* transcripts and repeats is reported in Fig. 1 (right panels). The relative abundance of rRNAs was higher in the G and S samples, likely due to partial degradation of large rRNAs that occurred during gland dissection and saliva collection. Reads mapping to miRNA precursors (hairpins) was high for the M, F and G samples (1.2 to 6.7 million reads) but rather small for the S sample (40,305 reads) (Fig. 1, Table 2). The proportion of reads mapping to other RNA types or to unannotated regions of the genome were comparable in the different samples, whereas those mapping to repeats were approximately 3 to 4 times higher in F (21.8%). The reason of this difference is unclear, however a similarly high frequency of reads mapping to repeats (>30%) was previously reported in adult *An. gambiae* females^53^.

**Table 2:** Reads mapping to precursor and mature miRNAs.

sample	AgamP4	hairpins (%)	mature (%)
S	7,758,653	40,305 (0.52)	28,475 (0.37)
G	21,069,482	1,244,151 (5.90)	1,188,871 (5.64)
F	28,771,027	5,628,344 (19.56)	5,514,013 (19.17)
M	26,466,637	6,766,708 (25.57)	6,624,225 (25.03)

Reads mapping to miRNA precursors were re-mapped to a collection of 438 mature (5p + 3p) miRNAs (Supplementary File S1). Altogether 28,475 reads from the saliva sample mapped to mature miRNAs, whereas 1.19, 5.51 and 6.62 million reads from the G, F and M samples, respectively, represented mature miRNAs (Table 2).

### miRNAs found in saliva, salivary glands, adult males and females

**Figure 2:**
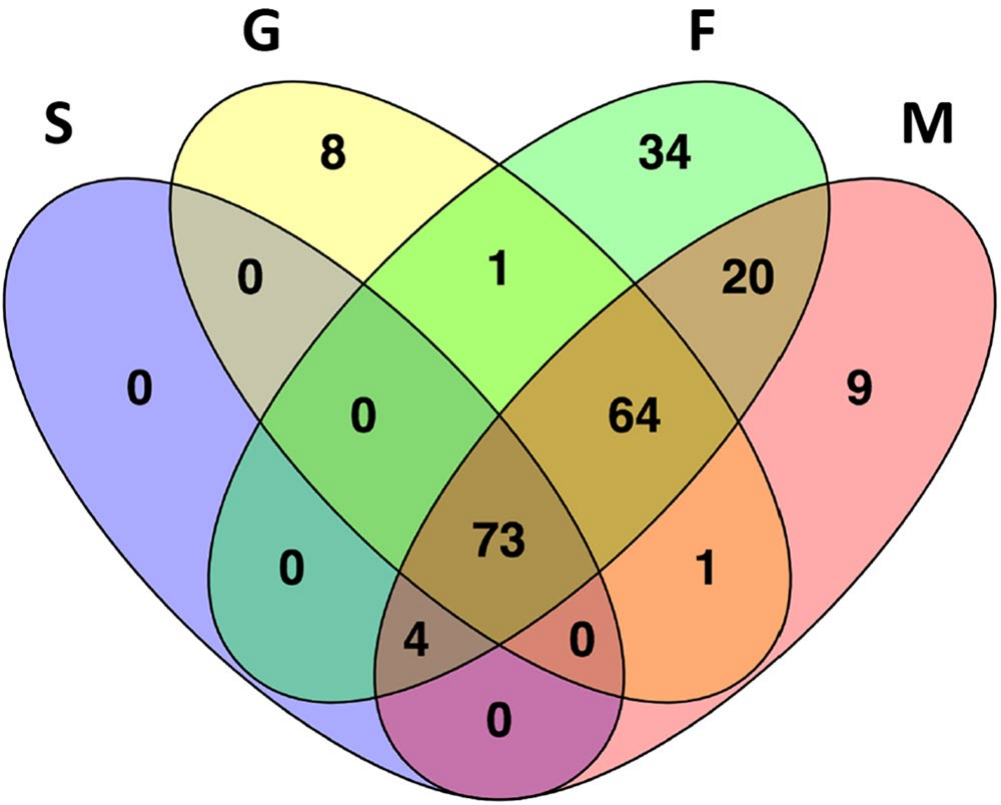
Distribution of the 214 mature miRNAs in the four samples analysed. The Venn diagram depicts the degree of overlap of the 214 miRNAs in the four samples: S (purple), G (yellow), F (green) and M (pink).

Overall, setting as a threshold for inclusion (i) the presence of counts in at least two replicates (three for the S sample) and (ii) a mean count per million (CPM) ≥ 3 in at least one of the four samples, we found *in silico* evidence for the expression of 214 mature miRNAs. Among these 178 were *An. coluzzii* orthologues of previously known *An. gambiae* miRNAs (miRBase^53–55^), and 87 of them matched 50 of the 57 *An. coluzzii* miRNA precursors previously identified by bioinformatics predictions^67^ and available in VectorBase^68^. The remaining 36 represent novel *An. coluzzii* and *An. gambiae* miRNAs. A list of these 214 miRNAs is reported in Supplementary File S2, where miRNAs were named using the prefix aco- followed by an identification code. For miRNAs previously described in *An. gambiae* we used the same identification; for those predicted by miRDeep* we used miR-N followed by a number; for those predicted by MapMi we kept as identification the name of the query miRNA. We then determined the subsets of miRNAs expressed in each sample and found 77 miRNAs in the saliva sample, with 66 supported by more than 500 mean CPM. As expected, a larger number of miRNAs was found in the other samples, with G, F and M including 147, 196 and 171 miRNAs, respectively (Supplementary File S2). Seventy-three miRNAs were common among the four samples and 51, instead, were unique to G, M or F samples (Fig. 2, Supplementary File S2).

### Novel miRNAs

**Figure 3:**
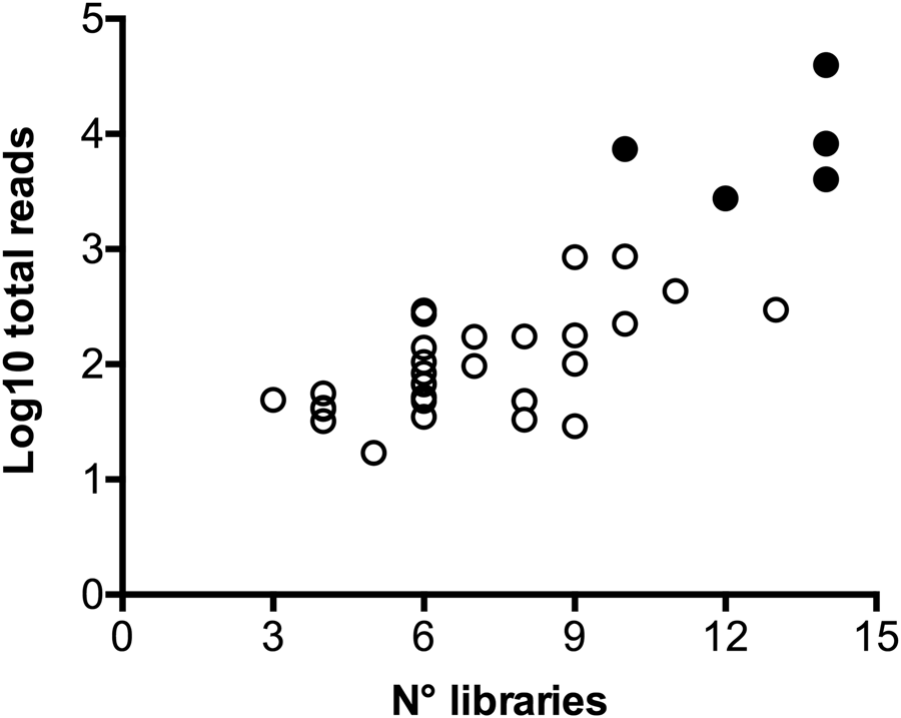
Abundance of the thirty-six novel miRNAs in the fourteen libraries. For each of the putative 36 novel miRNAs from *An. coluzzii* the number of total reads and the total number of libraries are reported. The five most abundant miRNAs, represented by >1000 reads, are shown with filled circles.

In the attempt to identify novel miRNAs, two complementary prediction tools were employed to search the AgamP4 assembly: miRDeep*^69^ and MapMi^70^, which exploit small RNA-Seq data and known miRNAs from other species, respectively. This way 99 putative mature miRNAs were predicted (Supplementary File S1). Overall, after mapping and applying the threshold for inclusion, 36 miRNAs appeared as *bona fide An. gambiae* and *An. coluzzii* novel miRNAs (Supplementary File S3). A large majority (30/36) was represented in at least six of the fourteen libraries and most of them (31/36) were of low abundance (Fig. 3). This is in agreement with previous studies on *An. gambiae* where the majority of novel miRNAs were found expressed at very low levels^58^. The remaining five miRNAs, instead, were well supported by counts (2,761-39,588) and CPM (103-58,697): these include two miRNAs matching the *Aedes aegypti* miR-71 stem-loop (MI0013440) and 3 completely novel miRNAs named aco-miR-N96, aco-miR-N56, aco-miR-N951. The secondary structure of the hairpins encoding these three abundant novel miRNAs from *An. coluzzii* are shown in Supplementary Figure S2.

Homologues of these 36 putative novel miRNAs were searched in the genomes of several anopheline and a few culicine mosquitoes as well as of other BFA and of the non-blood feeding Diptera *Drosophila melanogaster* and *Musca domestica*. The four miRNAs predicted by MapMi as well as aco-miR-N636 appeared widely conserved in mosquito species and also in a few other blood feeders (Supplementary Fig. S3). With the exception of aco-miR-N645, restricted to *An. gambiae* and *An. coluzzii*, all other miRNAs were found among members of the *An. gambiae* species complex but only occasionally in other species. Only three miRNAs showed a somehow wider distribution among mosquitoes, with aco-miR-N1044 exclusively found in anophelines and aco-miR-N149 and aco-miR-N1306 also found in culicine mosquitoes.

A subset of nine miRNAs of low (CPM < 20), medium (20 ≤ CPM < 100) and high (CPM ≥ 100) abundance in the F sample, were selected for validation by the Stem-loop Reverse-Transcription Polymerase Chain Reaction (slRT-PCR) technique^71^. Using small RNA from adult *An. coluzzii* females as template, clean amplifications of all selected miRNAs but one was obtained by slRT-PCR. The only exception was the least abundant aco-miR-N135 (7 CPM) that yielded an unspecific amplification product, most likely because of a not optimal primer design. An inverse correlation was found when mean CPM values were compared to Ct values as determined by the real time PCR amplification (Spearman r -0.833, p 0.015; Supplementary Fig. S4). Overall, these observations suggest that the large majority of the putative novel miRNAs identified here are indeed real and expressed in *An. coluzzi*.

### Differential expression analysis

**Figure 4:**
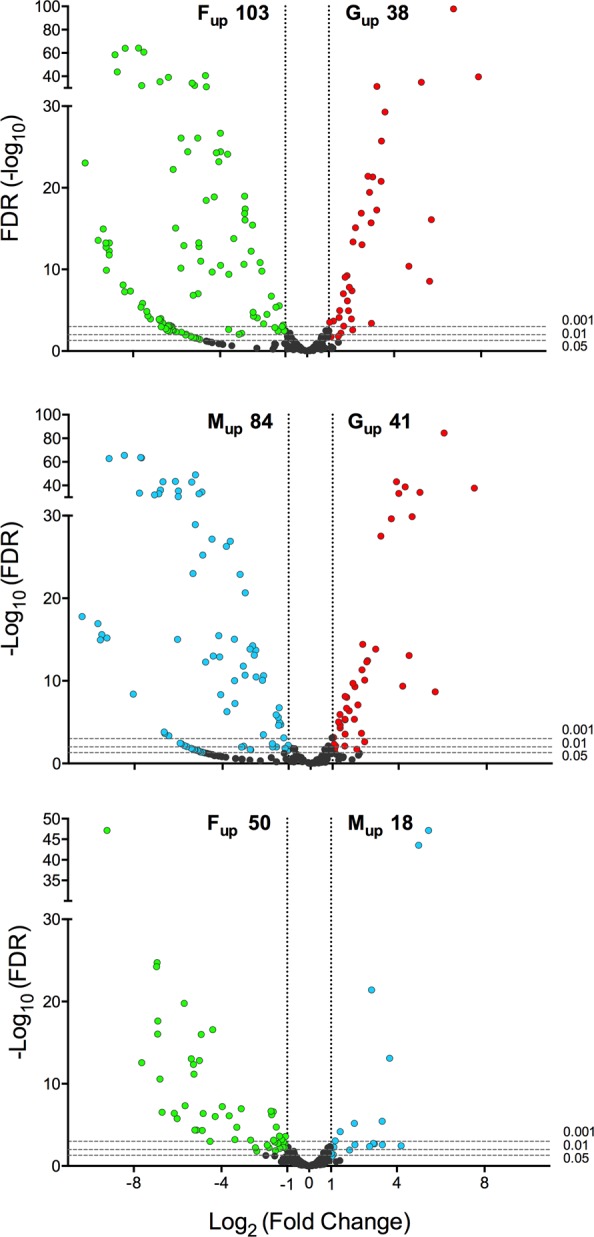
Differential abundance of miRNAs in *Anopheles coluzzii* salivary glands, adult females and adult males. The volcano plots show the differential abundance of miRNAs in the pairwise comparisons G-F (top), G-M (middle) and M-F (bottom). The log2 of fold change (FC) versus the negative log10 of false discovery rate (FDR) as calculated by the Fisher’s exact test are reported. Vertical dotted lines mark FC = 2, horizontal dashed lines mark FDR threshold equal to 0.05, 0.01 and 0.001. miRNAs with a FC > 2 and FDR < 0.05 in the different pairwise comparisons were considered as differentially expressed and are shown in red (upregulated in female salivary glands), green (upregulated in adult females) and blue (upregulated in adult males).

At first, differential expression (DE) analysis of mature miRNAs was performed on all four samples (S, G, F and M). This yielded a subset of miRNAs upregulated in the saliva sample; however, while ~40% of these miRNAs were well supported by counts, the remaining appeared differentially expressed despite the low or very low number of mapping reads. We assumed this was most likely due to the large difference in the number of reads mapping to miRNAs in the four samples. Therefore, to avoid any possible bias and make the downstream analysis more robust and reliable, we decided to (i) use the reads from the S sample just for assembling a catalogue of salivary miRNAs from *An. coluzzii* and (ii) repeat the DE analysis using only the G, F and M samples, which carried a comparable number of mapping reads. The mature miRNAs expression heatmap and the cluster analysis confirmed the overall quality of replicates, highlighting groups of miRNAs with specific profile signatures (Supplementary Fig. S5). Sample-specific miRNA enrichment was evaluated by pairwise comparisons between the three samples: fold change (FC) and false discovery rates (FDR) were calculated to provide statistical validation (Supplementary File S4). Using as threshold parameters FC > 2 and FDR < 0.05 we could identify subsets of miRNAs specifically enriched in adult female salivary glands, in adult females and in adult males. More in detail, the comparison G vs F highlighted 38 miRNAs enriched in female salivary glands and 103 significantly more abundant in adult females (Fig. 4, top panel), whereas the comparison G vs M pointed out the differential enrichment of 125 miRNAs, 41 upregulated in female salivary glands and 84 in adult males (Fig. 4, central panel). Finally, the comparison F vs M identified 68 sex-biased miRNAs that may play roles in sexual dimorphism: among these, 50 were more abundant in females and 18 in males (Fig. 4, bottom panel). The numbers of miRNAs found upregulated in the three pairwise comparisons according to different false discovery rates (FDR < 0.05, FDR < 0.01 and FDR < 0.001) are reported in Supplementary Table S1. Matching the two subsets of miRNAs upregulated in female salivary glands in the pairwise comparisons to F (38) and M (41) revealed that 30 miRNAs were common. These may be considered with good confidence as miRNAs specifically enriched in female salivary glands and, therefore, likely to play sex- and/or tissue-specific roles in salivary gland physiology and blood feeding.

### Asymmetric distribution of miRNAs in saliva and salivary glands of *Anopheles coluzzii*

**Figure 5:**
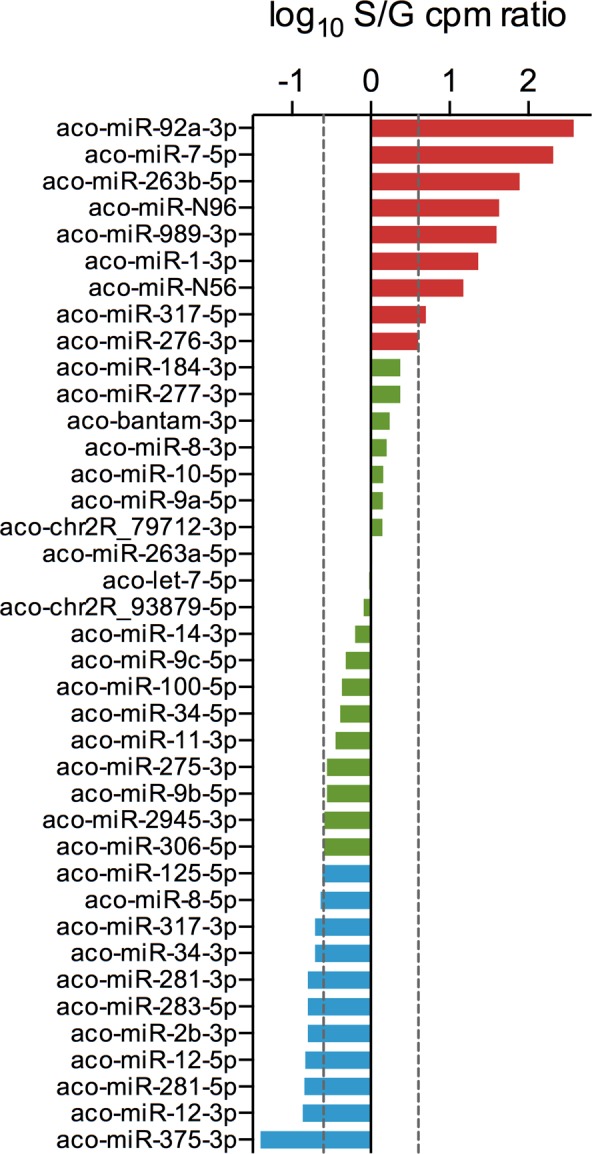
Asymmetric distribution of miRNAs in saliva and salivary glands. The mean CPM of the 30 most abundant miRNAs in saliva and salivary glands of *An. coluzzii* were compared and the S/G ratio calculated. For these 39 miRNAs the log10 of the S/G ratio is reported. miRNAs with S/G ratio > 4.0 or <0.25 are reported in red and light blue, respectively. In green are shown miRNAs whose S/G ratio was ≥0.25 or ≤4.0. Dashed lines mark the limits of 4-fold overexpression in saliva and salivary glands.

As mentioned above, the S sample was not included in DE analysis to avoid introducing any bias due to the low number of reads mapping to a group of miRNAs in the saliva sample. Nevertheless, we wondered whether miRNAs were or not symmetrically distributed between salivary glands and saliva. Therefore, we selected the 30 most abundant miRNAs from the S (CPM range 3,098-161,759) and G (CPM range 3,828-175,456) samples and, comparing the two lists, we found an overlap restricted to 21 miRNAs. When the S/G CPM ratio was calculated for the 39 miRNAs from the combined lists we found an asymmetric miRNA distribution between salivary glands and saliva. Indeed, 9 miRNAs had an S/G ratio > 4.00 and, therefore, appeared to be more abundant in saliva, whereas other 11 had an S/G ratio < 0.25 and were more represented in the salivary glands. The remaining 19, with S/G ratio ≤ 4.00 and ≥0.25, can be essentially considered as equally distributed (Fig. 5, Supplementary File S5). To verify if this distribution had a statistical support we examined the DE data obtained by edgeR for the pairwise comparison S-G during our initial analysis involving all four samples. The 9 miRNAs found over-represented in the S sample were supported by reliable mean CPM (range 3344–161759) and their differential distribution had very good statistical support (FDR values range 0.0057-3.61E-16); a similar situation was found for the 11 miRNAs over-represented in salivary glands (Supplementary File S5). These observations indicate that *An. coluzzii* saliva does not simply mirror salivary gland miRNA content and that a selected subset of miRNAs are preferentially conveyed to saliva.

### Target prediction

**Figure 6:**
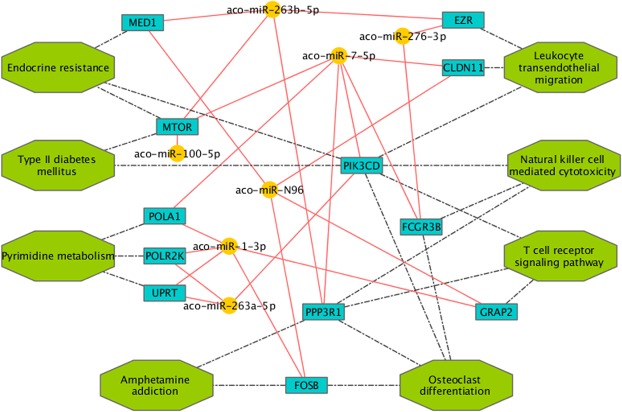
Target prediction analysis. Schematic representation of genes targeted by abundant miRNAs from *An. coluzzii* saliva and enriched categories. miRNAs are shown by yellow dots and targeted genes (blue boxes) indicated by solid red lines. Dotted lines connect genes to enriched KEGG pathways (green octagons).

MicroRNAs regulate gene expression by binding the 3′-UTR of mRNA targets^31,33,35^. Since mosquitoes inject saliva into their hosts during blood feeding, and considering that miRNAs in anopheline saliva may have evolved to play some role in vector-host interactions, we were wondering about potential targets of the most abundant miRNAs found in saliva. Based on the observation that the seed region (nucleotides 2–8 of the miRNAs) plays a crucial role in recognition and binding to mRNAs, as well as on a few other features as free energy or site accessibility, several bioinformatics tools employing different algorithms have been developed for target prediction^72,73^. However, the output of these software packages typically consists of a large number of potential targets, which makes the identification of genuine targets a difficult task. Moreover, predictions usually require some type of downstream validation, which can be challenging, especially in complex biological contexts as the interface between the mosquito and its vertebrate host. Nevertheless, the use of more than one bioinformatics tool and the selection of transcripts targeted by multiple miRNAs may help identify relevant pathways. Therefore, we employed three different bioinformatics tools (TargetSpy, miRanda and PITA) to predict human mRNA targets of the 8 most abundant miRNAs in saliva; only transcripts expressed in human skin according to transcriptomic (FPKM > 1.0)^74^ and proteomic^75^ studies were considered for the prediction. As a control the analysis was also performed employing 8 male and 8 female miRNAs not found in saliva or salivary glands. In order to avoid common biases, such as the different average 3′UTR length of genes belonging to different functional categories^76^, the pathway enrichment analysis of predicted miRNA targets was restricted to those mRNAs targeted by at least two salivary miRNAs and by none of the control miRNAs. KEGG pathway enrichment analysis, performed using the WebGestalt tool^77^, yielded some moderately enriched (p-value < 0.05, FDR > 0.05) but potentially meaningful pathways as T cell receptor signalling (hsa04660), leukocyte transendothelial migration (hsa04670) or natural killer cell mediated cytotoxicity (hsa04650) (Fig. 6). Among the predicted targets were: MTOR (Mammalian target of rapamycin) and PIK3CD (Phosphatidylinositol-4,5-bisphosphate 3-kinase catalytic subunit delta), which code for proteins playing central roles in important signalling cascades and regulating the function of diverse immune cells, including mast cells, neutrophils, T cells and B cells^78,79^; FCGR3B (Fc fragment of IgG receptor IIIb) that is expressed on neutrophils and whose product binds IgG and plays an active role in calcium mobilization and neutrophil degranulation^80^; PPP3R1 (Protein phosphatase 3 regulatory subunit B, alpha) also known as Calcineurin B that is involved in activation of transcription factors of the NFAT (Nuclear factor of activated T cells) family, which are key regulators of T-cell activation^81^; GRAP2 (GRB2-related adaptor protein 2), encoding a member of the Grb2 family of adaptor proteins implicated in the activation of lymphocyte-specific signalling pathways^82^; EZR (Ezrin), which encodes a protein linking plasma membrane to cytoskeleton and shown to play relevant roles in lymphocyte activation and migration^83^; FOSB that encodes a subunit of the transcription factor AP-1, which binds to promoters of early response and inflammatory genes. Overall, target prediction analysis suggest that the most abundant miRNAs in the saliva of *An. coluzzii* have the potential to target human genes involved in inflammatory and immune responses.

### miRNAs from *Anopheles coluzzii* saliva mimic human miRNAs

We also wondered if miRNAs found in *An. coluzzii* saliva may mimic known human miRNAs. Searching miRBase it was found that 11 out of the 20 most abundant miRNAs from *An. coluzzii* saliva were identical or almost identical to human miRNAs (Table 3). Interestingly, these human miRNAs have several targets among genes coding for chemokines, cytokines, chemokine receptors, mitogen-activated protein (MAP) kinases, transcription factors and other mediators crucially affecting inflammatory and immune responses by acting on the NF-kB pathway, Toll-like receptors (TLRs) signalling cascades and inflammasome activation (Supplementary Table S2). A few paradigmatic examples are shortly discussed below whereas a more extensive, though not exhaustive list, can be found in Supplementary Table S3. Hsa-miR-7-5p was shown to down-regulate the NF-kB pathway both directly, by targeting the NF-kB subunit RelA^84^, and indirectly, by acting on RNF183, an ubiquitin ligase promoting the degradation of the NF-kB inhibitor IkBα^85^. Hsa-miR-1-3p targets CCL2^86^, an inflammatory chemokine that is a powerful chemotactic factor involved in the recruitment of monocytes, memory T cells and natural killer (NK) cells to sites of inflammation produced either by tissue injury or infection^87^. Hsa-miR-184 downregulates NFAT1, a key transcription factor that controls the expression of a wide array of cytokines (IFNγ, GM-CSF, IL-3, IL-4, IL-2, TNFα) and plays a crucial role in the initiation of Th1 immune response^88^. Hsa-miR-92a-3p has, among other targets, also CCL8^89^, a chemokine displaying chemotactic activity for monocytes, lymphocytes, basophils and eosiniphils. Hsa-miR-200b-5p was shown to target MyD88 in macrophages, a key mediator of TLR signalling and NF-kB activation^90^. Finally, hsa-miR-125b-5p may act on two key transcription factors in B cell differentiation as the interferon regulatory factor 4 (IRF4) and PRDM1^91^. These observations reinforce the target prediction analysis reported above clearly pointing to the ability of abundant miRNAs from *An. coluzzii* saliva to target human inflammatory and immune responses.

**Table 3:** Human orthologues among the 20 most abundant saliva miRNAs.

*An. coluzzii* ID	nt	*H. sapiens* ID	seed	mm	alignment
aco-miR-276-3p	22	—	—	—	—
aco-miR-263a-5p	24	—	—	—	—
**aco-miR-7-5p**	23	**hsa-miR-7-5p**	**Y**	0	1-23/1-23
**aco-miR-1-3p**	22	**hsa-miR-1-3p**	**Y**	1	1–22/1–21
**aco-miR-100-5p**	22	**hsa-miR-100-5p**	**Y**	0	1–22/1–22
**aco-miR-184-3p**	21	**hsa-miR-184**	**Y**	0	1-21/1–21
aco-miR-N96	23	—	—	—	—
aco-miR-263b-5p	21	—	—	—	—
aco-miR-8-3p	23	hsa-miR-141-3p	Y/N	2	1–21/1–21
**aco-let-7-5p**	21	**hsa-let-7a-5p**	**Y**	1	1–21/1–21
aco-bantam-3p	22	—	—	—	—
**aco-miR-92a-3p**	22	**hsa-miR-92a-3p**	**Y**	1	1–22/1–22
**aco-miR-8-5p**	22	**hsa-miR-200b-5p**	**Y**	2	1–22/1–22
**aco-miR-34-5p**	20	**hsa-miR-34c-5p**	**Y**	2	2–20/2–20
aco-miR-N56	23	—	—	—	—
aco-miR-10-5p	21	hsa-miR-10a-5p	Y/N	0	1–21/2–22
aco-miR-14-3p	21	—	—	—	—
**aco-miR-125-5p**	22	**hsa-miR-125b-5p**	**Y**	0	1–22/1–22
aco-miR-306-5p	22	—	—	—	—
aco-chr2R_79712-3p	22	—	—	—	—

nt, miRNA length; seed, fully (Y) or partially (Y/N) conserved seed (nt 2–8); mm, number of mismatches in the aligned region; alignment, nucleotide position of aligned *An. coluzzii* to *H. sapiens* miRNAs. Human orthologues of *An. coluzzi* miRNAs with fully conserved seeds are in bold.

### Comparison of saliva miRNAs among different species

Saliva miRNA repertoires have been previously studied in a few BFAs, specifically the mosquitoes *Ae. aegypti* and *Ae. albopictus* and the tick *I. ricinus*^40,41^. When we compared the top 30 miRNAs from *An. coluzzii* saliva to the 30 most abundant miRNAs found in the saliva of *Ae. aegypti*, *Ae. albopictus* and *I. ricinus* we found that 17 were also present in the saliva of *Aedes* mosquitoes and 16 in *I. ricinus* saliva; more specifically, 15 miRNAs were common to the three mosquito species and 10 miRNAs to both mosquitoes and the hard tick (Table 4, Supplementary File S6). We also mined a collection of miRNA profiles from the saliva of 46 healthy human subjects^39^ to verify if the 11 miRNAs from *An. coluzzii* saliva mimicking human miRNAs (Table 3) were also present in human saliva. We found that only 3 miRNAs (hsa-miR-141-3p, hsa-let-7a-5p, hsa-miR-92a-3p) were among the top 50 miRNAs in human saliva, other 3 were completely absent (hsa-miR-1-3p, hsa-miR-184, hsa-miR-200b-5p) and the remaining were present at low abundance (rank 67 to 248; Supplementary File S6 and Table 4). Overall, these observations support the idea that the presence of miRNAs in *An. coluzzii* saliva, at least as far as the most abundant miRNAs are concerned, it is most likely not serendipitous and that they may play some functional role.

**Table 4:** Conservation of the top 30 miRNAs from *An. coluzzi* saliva.

*An. coluzzii ID*	*H. sapiens* ^a^	*Ae. aegypti* ^b^	*Ae. albopictus* ^b^	*I. ricinus* ^c^	*B. malayi* ^d^	*H. polygyrus* ^d^
aco-miR-276-3p		✔	✔	✔		
aco-miR-263a-5p		✔	✔	✔		✔
**aco-miR-7-5p**					✔	
**aco-miR-1-3p**					✔	✔
**aco-miR-100-5p**		✔	✔	✔	✔	✔
**aco-miR-184-3p**		✔	✔			
aco-miR-N96						
aco-miR-263b-5p						
**aco-miR-8-3p**	✔	✔	✔	✔		
**aco-let-7-5p**	✔	✔	✔	✔	✔	✔
aco-bantam-3p		✔	✔		✔	✔
**aco-miR-92a-3p**	✔			✔	✔	
**aco-miR-8-5p**		✔	✔	✔		
**aco-miR-34-5p**		✔	✔	✔	✔	
aco-miR-N56						
**aco-miR-10-5p**		✔	✔	✔		
aco-miR-14-3p		✔	✔			
**aco-miR-125-5p**		✔	✔	✔		✔
aco-miR-306-5p				✔		
aco-chr2R_79712-3p						
aco-miR-281-3p		✔	✔			
aco-miR-281-5p		✔	✔			
aco-miR-317-3p		✔	✔	✔		
**aco-miR-9a-5p**				✔	✔	✔
aco-chr3R_160384-3p				✔		
aco-miR-277-3p		✔	✔			
aco-miR-275-3p				✔		
aco-miR-989-3p		✔				
aco-miR-317-5p						
aco-chr2R_93879-5p			✔	✔	✔	
total	3	17	17	16	9	7
human	3	8	8	9	7	5

The presence among the top 50 in human saliva, the top 30 in the saliva of *Ae. aegypti*, *Ae. albopictus* and *I. ricinus* and the top 30 exosomal miRNAs from the parasitic nematodes *Brugia malayi* and *Heligmosomoides polygyrus* are reported. The total number of conserved miRNAs and the number of those homologous to human miRNAs are shown at the bottom. *An. coluzzi* miRNAs mimicking human miRNAs are in bold. Extended data are reported in Supplemental File S6. 1. Yeri A. *et al*. 2017 Sci Rep 7:44061; 2. Maharaj P.D. *et al*. 2015 PLoS Negl Trop Dis 9:e0003386; 3. Hackenberg M. *et al*.^40^ RNA 23(8):1259–1269; 4. Zamanian M. *et al*. 2015 PLoS Negl Trop Dis 9:e0004069; 5. Buck A.H. *et al*. 2014 Nat Commun 5:5488.

Interestingly, the parasitic nematodes *Brugia malayi* and *Heligmosomoides polygyrus* (which infect humans and mice, respectively) were shown to secrete exosomal vesicles enriched in miRNAs identical/homologous to host miRNAs with known immunomodulatory roles. These exosomes could be internalized by target cells *in vitro* and *H. polygyrus*-derived exosomes could suppress innate immunity *in vivo* when administered to mice^51,52^, suggesting they may play a role in host manipulation. Therefore, we also compared the 30 most abundant exosomal miRNAs from these nematodes to the top 30 from *An. coluzzi* saliva and found 9 and 7 miRNAs in common with *B. malayi* and *H. polygyrus*, respectively (Table 4, Supplementary File S6).

## Discussion

Transcriptomic, proteomic and genomic studies performed in the last decades shed light on the complexity and functions of salivary protein repertoires of hematophagous arthropods. Saliva of BFAs carries hundreds of proteins whose anti-haemostatic, anti-inflammatory and immunomodulatory activities have a highly adaptive value in hematophagy providing blood feeders with the ability to manipulate host responses at the feeding site^4,6,23^. However, despite the considerable progress, the understanding of the complex interactions taking place at the vector-host interface is still limited since we completely ignore the function of a rather large fraction of BFA salivary proteins and we just started getting deeper insights into the role of saliva in transmission of vector-borne pathogens^27,30,92–96^. Moreover, the finding of miRNAs in the saliva of *Aedes* mosquitoes^41^ and *Ixodes* ticks^40^ suggests that saliva of BFA may have a more complex composition than initially predicted.

To get insights into miRNA composition in the saliva of anopheline mosquitoes we performed a small RNA-Seq study on adult female salivary glands and saliva of the African malaria vector *An. coluzzii*. The number of reads mapping to mature miRNAs for the S sample was limited, most likely because of the difficulty of getting large amounts of saliva and to the paucity of small RNA content: indeed, comparable number of reads mapping to *Aedes albopictus* (12,075) and *Aedes aegypti* (298,283) miRNAs were obtained in the only study presently available on miRNAs from mosquito saliva^41^. While the most abundant miRNAs in the S sample were supported by a reliable number of counts, the least abundant had sometime less than 50 reads mapped (Supplementary File S2, worksheet aco_S_77). For this reason, to avoid introducing any bias, we used the S sample just to assemble a saliva catalogue of *An. coluzzii* miRNAs and restricted differential expression analysis to the G, F and M samples. Overall, evidence for the expression of 214 mature miRNAs was obtained, with 36 being putative novel miRNAs of *An. coluzzii* and *An. gambiae*. Almost all of these 36 novel miRNAs appeared conserved among members of the *An. gambiae* species complex, with a few also present in other anopheline and culicine mosquitoes and occasionally in other blood feeders. Using PCR amplification we provided evidence of expression for a subset of these *bona fide* novel miRNAs and found very good correlation between RNAseq and real time PCR data (Supplementary Fig. S4). These observations suggest that most of these miRNAs are real and mainly expressed at low levels in members of the *An. gambiae* species complex.

Differential expression analysis identified 38 miRNAs enriched in female salivary glands as compared to adult females: these may regulate endogenous genes involved in salivary gland physiology and/or blood feeding. Ten of these G-enriched miRNAs were also abundant in saliva (among the top 30) and, therefore, are likely to be injected into the vertebrate skin during blood feeding with the potential to target host genes at the biting site. In addition, 68 miRNAs appeared sex-biased, with 50 enriched in females and 18 in males, and they are expected to be implicated in sexual dimorphism. A list of miRNAs differentially abundant in the samples analysed in this study is provided in Supplementary Table S4 and may represent a useful starting point for further investigations.

One of the main goals of our experimental design was to enquire whether salivary glands and saliva from *Anopheles* mosquitoes have an identical or somewhat different miRNA composition. Indeed, an enrichment for selected miRNAs in saliva, as compared to salivary glands, has been previously reported in the hard tick *Ixodes ricinus*^40^; however, it was unknown if this was the case also for mosquitoes since the *Aedes* study of Maharaj and collaborators (2015) was focused on the effect of chikungunya virus infection and did not include a parallel analysis of salivary glands. The *An. coluzzii* saliva miRNA catalogue comprised 77 mature miRNAs, a number which is comparable to what previously found in *Ae. albopictus* (67 miRNAs) and *Ae. aegypti* (103 miRNAs)^41^. Although, as mentioned above, the least represented miRNAs had small number of counts, the 30 most abundant (top 30) were found in all five S replicates (29 cases out of 30) and appeared well supported by number of reads (range 52–4291) and CPM (range 3097–161,759) (Supplementary File S2). Moreover, since the five S replicates were processed in two batches and exhibited an overall lower correlation (see heatmap, Supplementary Fig. 1), we also compared the lists of the top 30 miRNAs from the two saliva sets and found a 77% overlap. When the relative abundance of the top 30 miRNAs in salivary glands and saliva of *An. coluzzii* was compared, we found groups of miRNAs overrepresented (FC > 4) in S or in G and others roughly equally distributed (Fig. 5), which indicated that specific miRNAs may be preferentially directed toward the secretory pathway or retained in salivary glands. It should be pointed out that asymmetric distribution of miRNAs between source cells and their exosomes has been previously reported and that multiple mechanisms appear to underlie their sorting into exosomes^43^, including recognition of exomotifs by RNA Binding Proteins^97^ or non-templated nucleotide addition (NTA) at the 3′-end of the miRNAs^98^. More specifically, 3′-end uridylated miRNA isoforms appeared overrepresented in exosomes and 3′-end adenylated in their mother cells^98^. We could not find evidence of enrichment of previously described exomotifs^97^ in *An. coluzzii* miRNAs abundant in saliva. However, when we determined the fraction of U and A non-templated addition to the 3′-end of miRNAs from saliva and non-saliva samples, a higher proportion of uridylation in saliva miRNAs was found (Supplementary Fig. S6). A similar situation was reported for saliva miRNAs from the tick *I. ricinus* and interpreted as an indirect evidence of exosomal origin of saliva miRNAs^40^, which may be the case for anopheline mosquitoes as well.

Comparing the top 30 saliva miRNAs from *An. coluzzii*, *Ae. aegypti*, *Ae. albopictus* and *I. ricinus*, it was found that 15 miRNAs were evolutionary conserved between *Anopheles* and *Aedes* mosquitoes and 10 miRNAs were common to both mosquitoes and the hard tick (Table 4, Supplementary File S6). Moreover, since 11 *An. coluzzii* saliva miRNAs were essentially identical to human endogenous miRNAs, we also wondered if they were abundant in human saliva. However, when a relatively large collection of human saliva miRNA profiles^39^ was searched, only 3 of these miRNAs were included among the top 50 miRNAs in human saliva (Supplementary File S6, Table 4). Furthermore, the 30 most abundant saliva miRNAs from *An. coluzzii* were also compared to exosomal miRNAs from the parasitic nematodes *B. malayi* and *H. polygyrus*. These miRNAs, many of which mimic host miRNAs, were previously suggested to play roles in manipulation of host innate immunity^51,52^. Interestingly, *An. coluzzi* saliva shared 9 miRNAs with *B. malayi* and 7 miRNAs with *H. polygyrus* (Table 4, Supplementary File S6).

The asymmetric distribution and the conservation of sets of miRNAs in the saliva of blood feeding arthropods (and parasitic nematodes) suggest a possible functional role for saliva miRNAs and, obviously, this leads to the question of potential host targets. Setting strict criteria to minimize false positives, and selecting only targets predicted by multiple tools, we could identify some potentially meaningful candidate target genes involved in human immune and inflammatory responses (Fig. 6). Moreover, the presence of human homologues among the most abundant miRNAs in *An. coluzzi* saliva came as an unexpected help for target analysis. Indeed, a quite large body of literature reporting experimentally validated human miRNA targets is available, and these 11 human miRNAs were found to target several genes and pathway involved in human inflammatory and immune responses (Supplementary Tables S2–S3). We believe that these observations point to the fascinating hypothesis that miRNAs in mosquito saliva, perhaps enclosed within exosomes, may target vertebrate host cells involved in immune and inflammatory responses.

The question whether miRNAs from body fluids play biological functions is still debated^44,45^; it has been calculated that miRNAs expressed below ~100 copies per cell have little regulatory capacity^99^ and that the concentration of extracellular miRNAs may be too low to exert *in vivo* effect^100^. However, there are several indications that exosomal miRNAs can be transferred *in vitro* to recipient cells where they can affect gene expression^44,101^ and the majority of miRNAs from human saliva was shown to be concentrated in exosomes^102^. Moreover, circulating exosomal miRNAs from adipose tissue have been recently shown to regulate gene expression in the liver^46^, reinvigorating the hypothesis of an *in vivo* functional role of exosomal miRNAs. According to this scenario, BFAs may manipulate host responses not only using salivary proteins but also taking advantage of salivary miRNAs. Haemostatic, inflammatory and immune responses of vertebrates are known to be both complex and redundant, and, to efficiently deal with their hosts, BFAs evolved a cocktail of salivary proteins of similar redundancy and complexity^4,23^. However, while salivary proteins may evoke in vertebrate hosts inactivating antibody responses that may affect blood feeding efficiency, miRNAs are not immunogenic and, therefore, would provide hematophagous arthropods with a precious additional tool. The evolutionary advantage of such a combined strategy seems obvious for ticks, which can stay attached to their hosts for days, or for parasitic nematodes, that establish long-term interaction with the host. On the contrary, the benefit does not appear equally evident for blood feeders as mosquitoes or sand flies that take their blood meals in a timescale of minutes, not compatible with the time of action of miRNAs. Nevertheless, even if not providing an immediate reward to the individual, the modulation of the host antibody response to salivary proteins (which could be achieved for example by targeting antigen presenting cells) may represent a longer-term evolutionary advantage for the species. Providing further evidence that miRNAs from saliva of BFAs play a physiological role in host manipulation (and perhaps in pathogen transmission) will require further experimentation that may be challenging considering the intricacy of the vector-host-pathogen relationships. Anyhow, we believe that our study contributes to a better understanding of function and complexity of blood feeding arthropod saliva and opens perspectives for novel investigations.

## Methods

### Mosquito rearing and sample collection

*An. coluzzii* mosquitoes (Xag, 2 R+, 2 L+, 3 R+, 3 L+; colony originally collected in Cameroon), formerly known as *An. gambiae* M molecular form^103^, were reared under standard insectary conditions (28 ± 1 °C, 60–70% humidity, 14:10 hours light:dark photoperiod). Adults 2–6 days post-emergence (dpe) were used for all the experiments reported here. Salivary glands were dissected in Phosphate Buffered Saline (PBS), collected in RNAlater (Sigma-Aldrich, R0901), kept overnight at 4 °C and then stored at −20 °C until needed. Adult males and females were immersed in RNAlater and stored as above. Saliva was collected from adult females as previously described^104,105^ with some modifications. Briefly, mosquitoes were deprived of wings and legs, immobilized on double-stick tape on a microscope slide, and their proboscis was inserted into a 10 µl pipet tip filled with 2–3 µl of RNAlater/PBS (50% v/v). Pilocarpine (1% w/v in PBS) was applied to the thorax and mosquitoes were left salivating for 10–15 minutes; afterwards the RNAlater/PBS solution containing saliva was ejected into eppendorf tubes containing 30 µl of RNAlater. Saliva samples (batches of saliva from 20 to 70 mosquitoes) were stored at −20 °C until used for RNA extraction. All samples were collected in low-binding tubes (Sigma-Aldrich, Z666505).

### RNA extraction, library preparation and RNA-sequencing

In a pilot experiment, performed on duplicates, small RNA was extracted from saliva collected from 97 and 98 mosquitoes, respectively, using the miRNeasy Serum/Plasma kit (QIAGEN). In a second experiment 80 salivary glands, 5 adult females, 5 adult males and saliva collected from 126–128 mosquitoes (all in triplicates) were used for small RNA extraction according to the miRNeasy Micro kit (salivary glands, adult males and females) and the miRNeasy Serum/Plasma kit (saliva) protocols (QIAGEN). miRNA validation was performed using as template the small RNA fraction (<200 nt) extracted from adult *An. coluzzii* females using the miRNeasy Micro kit. Concentration and purity of small RNA were evaluated determining the absorbance at 260 and 280 nanometers by a BioTek SynergyHT (Take3 Module). RNA quality control and libraries preparation were performed by the EMBL Genomic Core Facility (EMBL, Heidelberg, DE). RNA quality and integrity was assessed using an Agilent 2100 Bioanalyzer (Agilent Technologies). Small RNA libraries were prepared using the TruSeq Small RNA Sample preparation kit (Illumina). Fifty base pair, single end sequencing was performed on an Illumina HiSeq2000 platform. An Illumina MiSeq platform (read length 75, single end sequencing) was used for the pilot saliva duplicated samples.

### Reads Mapping

Raw reads were first quality control checked by FastQC^106^ and then trimmed using cutadapt 1.9.1^107^ to remove 3′ adapters and discard reads shorter than 14 nucleotides. Processed reads from each sample were mapped to the *An. gambiae* AgamP4 genome assembly (version v2.00, downloaded from VectorBase^108,109^) using Bowtie^110^ (-n 0 -l 18 -e 80). Indeed, although the *An. coluzzii* genome has been sequenced (Mali-NIH strain, AcolM1 assembly), the reference *An. gambiae* PEST genome was preferred for several reasons. First, *An. gambiae* and *An. coluzzii* only recently have been classified as different species^103^, being formerly considered as incipient species and known as *An. gambiae* S and M molecular forms, respectively. Second, genome assemblies and annotations are very different for quality and accuracy: AcolM1 consists of 10,521 scaffolds with an N50 of 4,437 Kb, whereas AgamP4 includes 8 scaffolds with an N50 of 49,364 Kb and with the assembly mapped to chromosomes^18^. Finally, the reference *An. gambiae* PEST genome is actually a chimera of S and M molecular forms^111^. Anyhow, we also attempted using AcolM1 but, as expected, the number of reads mapping to the genome was 10% to 26% lower (depending from the sample), which further supported our choice. Reads aligned to AgamP4 were then mapped (-n 0 -l 18 -e 80–norc) to a collection of *An. gambiae* rRNA sequences obtained from VectorBase by the BioMart tool. After subtraction of ribosomal RNAs the remaining reads were mapped (-n 0 -l 18 -a–best–strata -e 80–norc) to a list of miRNA precursors and other non coding RNAs from *An. gambiae* (including tRNAs, snoRNAs, snRNAs, 7SL, 7SK and RNase P) and finally to *An. gambiae* transcripts and repeats downloaded from VectorBase (-n 0 -l 18 -a–best–strata -e 80). The list of miRNA precursors included a total of 273 hairpins (Supplemental File 1): among these 175 were previously known *An. gambiae* hairpins (66 retrieved from miRBase release 21, 59 from^53^, 41 from^54^, 9 from^55^) and the remaining 98 were predicted (see below). The collection of mature miRNAs consisted of 438 miRNAs (5p + 3p, Supplemental File 2): 339 previously known *An. gambiae* miRNAs (131 from miRBase, 118 from^53^, 81 from^54^ and 9 found by^55^) and 99 additional predicted miRNAs (see below).

### Prediction of novel miRNAs by miRDeep* and MapMi

Reads from all samples which did not map to *An. gambiae* rRNAs or known small RNAs were combined and used to predict novel miRNAs by employing the miRDeep* software^69^. A cutoff score of 0 was used to select the most reliable novel miRNA predictions. We also used MapMi^70^ to map miRNAs from other species (arthropods, human and viruses retrieved from miRBase release 21) to the *An. gambiae* genome (threshold 25, max mature mismatch 3). This way we compiled a set of loci potentially orthologous to miRNAs validated in other species. miRNAs found by MapMi whose genomic coordinates overlapped those of known or miRDeep*-predicted miRNAs were identified through the BEDTools software^112^ and discarded. Secondary structure predictions and minimal free energy calculations were performed using RNAfold^113^.

### miRNA validation by real-time PCR amplification

Validation of a subset of the putative novel miRNAs identified in this study was performed by the Stem-loop Reverse-Transcription Polymerase Chain Reaction (slRT-PCR) technique^71^ using as template small RNA from adult *An. coluzzii* females. First-strand cDNA was generated in a 20 µL reaction volume from 1 µg of small RNA using the SuperScript II Reverse Transcriptase (Invitrogen) according to manufacturer’s instruction and specific stem-loop primers (0.1 µM). Nine miRNAs were selected for validation according to their CPM values and choosing three miRNAs for each of the tree following abundance categories: (i) high (CPM ≥ 100; aco-miR-N96, aco-miR-N56, aco-miR-N951; range 103–489), (ii) medium (20 ≤ CPM < 100; aco-miR-N966, aco-miR-N149, aco-miR-N629; range 23–73) and (iii) low (CPM < 20; aco-miR-N1044, aco-miR-N645, aco-miR-N135; range 7–17). Real time PCR amplifications were performed in a final volume of 20 µl including 2X PowerUP SYBR Green Master Mix (Applied Biosystem), specific forward and universal reverse primers (1 µM each) and 2 µl of the specific first strand cDNA reaction. Amplification was as follows: initial holding stage of 2 min at 50 °C and 2 min at 95 °C followed by 40 cycles (30 sec. 95 °C, 1 min 60 °C). Melting curves were obtained for each miRNA to verify for the absence of unspecific amplification products with detection steps every 0.3 °C. All RT-qPCR reactions were performed in biological and technical triplicates. A list including the specific stem-loop and reverse primers, as well as the universal primers is provided in Supplementary Table S5.

### Conservation of novel miRNAs

Putative orthologues of novel miRNAs were searched using the BLAST tool at the VectorBase web site^109^. The genomes of several anopheline, a few culicine mosquitoes (*Aedes aegypti*, *Aedes albopictus* and *Culex quinquefasciatus*), the sand flies *Phlebotomus papatasi* and *Lutzomyia longipalpis*, the tsetse fly *Glossina morsitans*, the stable fly *Stomoxys calcitrans*, the bugs *Rhodnius prolixus* and *Cimex lectularius*, the human body louse *Pediculus humanus*, the tick *Ixodes scapularis* and of the non blood feeding Diptera *Drosophila melanogaster* and *Musca domestica* were searched. A miRNA was considered as putatively conserved in a given species if the BLASTn search yielded (i) ≥70% identity over ≥70% of the length of miRNA precursors and/or (ii) ≥90% identity over the entire length and fully conserved seed sequence for mature miRNAs.

### Quantification and differential expression of miRNAs

Read counts for each *An. gambiae* small RNA were computed from SAM files using a Python custom script. Reads with multiple highest score mappings were discarded. Expression values were calculated as count per millions (CPM) and used for sample clustering. Reads mapping to precursor miRNAs were assigned to mature miRNAs based on their mapping position; an overhang of maximum 3 nucleotides for each side of the mature form was tolerated. Differential expression analysis of mature miRNAs with 1 CPM in at least three samples was performed using glmFIT and glmLRT functions provided by the edgeR software package^114,115^. Fold change (FC) and false discovery rates (FDR) were calculated to provide statistical validation (Supplementary File S4).

### 3′-end non-templated U and A addition

To identify reads representing 3′-end non-templated additions of uridine and adenine residues, miRNAs we first searched for tags carrying at least one substitution following the 18 nt long seed region. Thereafter, for reads presenting these mismatches, the subsequence beginning at the first substitution site was evaluated for the presence of only Ts or As.

### Target analysis

Host genes putatively targeted by *An. gambiae* miRNAs were searched using the miRNAconsTarget program from sRNAtoolbox^116^, which employs the prediction software TargetSpy, miRanda and PITA^117–119^. The 8 most abundant miRNAs found in *An. coluzzii* saliva (aco-miR-276-3p, aco-miR-263a-5p, aco-miR-7-5p, aco-miR-1-3p, aco-miR-100-5p, aco-miR-184-3p, aco-miR-N96, aco-miR-263b-5p) were used as query. Eight male (aco-chrX_329630-5p, aco-miR-2c-5p, aco-miR-10367-5p, aco-miR-219-5p, aco-miR-10375a-3p, aco-chr3L_119935-5p, aco-miR-10372a-5p, aco-miR-10370-3p) and 8 female (aco-miR-989-5p, aco-chr3R_150582-5p, aco-miR-10362-5p, aco-miR-N420, aco-chr3L_130625-5p, aco-miR-N148, aco-chr2L_42099-5p, aco-miR-10359-5p) miRNAs not found in saliva or salivary glands were used as a control set (Supplementary File S2). Only miRNA-mRNA interactions predicted by all three programs were taken into consideration. Prediction was done using 3′UTRs (>30 nt) of transcripts expressed in human skin^74,75^, which were downloaded from Ensembl^120^ using the BioMart tool^121^. The corresponding genes were provided as background for the KEGG pathway enrichment analysis that was performed using the WebGestalt tool^77^.

## Supplementary information

Supplementary Information

Supplementary file S1

Supplementary file S2

Supplementary file S3

Supplementary file S4

Supplementary file S5

Supplementary file S6

## Data Availability

Small RNA-Seq data have been submitted to the NCBI GEO repository with accession number GSE:120658 (https://www.ncbi.nlm.nih.gov/geo/query/acc.cgi?acc = GSE120658) and will be publicly released upon manuscript acceptance. Other data generated during this study have been included as Supplementary Information.
