# Supplementary material for: MicroRNAs from saliva of anopheline mosquitoes mimic human endogenous miRNAs and may contribute to vector-host-pathogen interactions
Source: Sci Rep. 2019 Feb 27;9:2955. doi: 10.1038/s41598-019-39880-1 (PMC6393464; doi:10.1038/s41598-019-39880-1)
Supplement: Supplementary file 1 — Supplementary Information [file 41598_2019_39880_MOESM1_ESM.pdf]

Supplementary Figures and Tables

MicroRNAs from saliva of anopheline mosquitoes mimic human endogenous miRNAs and may contribute to vector-host-pathogen interactions

Bruno Arcà[1*], Alessio Colantoni[2], Carmine Fiorillo[1], Francesco Severini[3], Vladimir Benes[4], Marco Di Luca[3], Raffaele A. Calogero[5] and Fabrizio Lombardo[1]

1. *Department of Public Health and Infectious Diseases, "Sapienza" University, Piazzale Aldo Moro 5 – 00185 Rome, Italy.*
2. *Department of Biology and Biotechnology, "Sapienza" University, Piazzale Aldo Moro 5 – 00185 Rome, Italy.*
3. *Department of Infectious Diseases, Istituto Superiore di Sanità, Viale Regina Elena 299, 00161 Rome, Italy.*
4. *Genomics Core Facility, European Molecular Biology Laboratory, Meyerhofstrasse 1, 69117 Heidelberg, Germany.*
5. *Department of Molecular Biotechnology and Health Sciences, University of Turin, Via Nizza 52, 10126 Turin, Italy.*

***Corresponding author**:

Bruno Arcà, Department of Public Health and Infectious Diseases – Division of Parasitology, Sapienza University, Piazzale Aldo Moro 5, 00185 Rome, Italy. Tel: +39 06 4991 4413. E-mail: bruno.arca@uniroma1.it

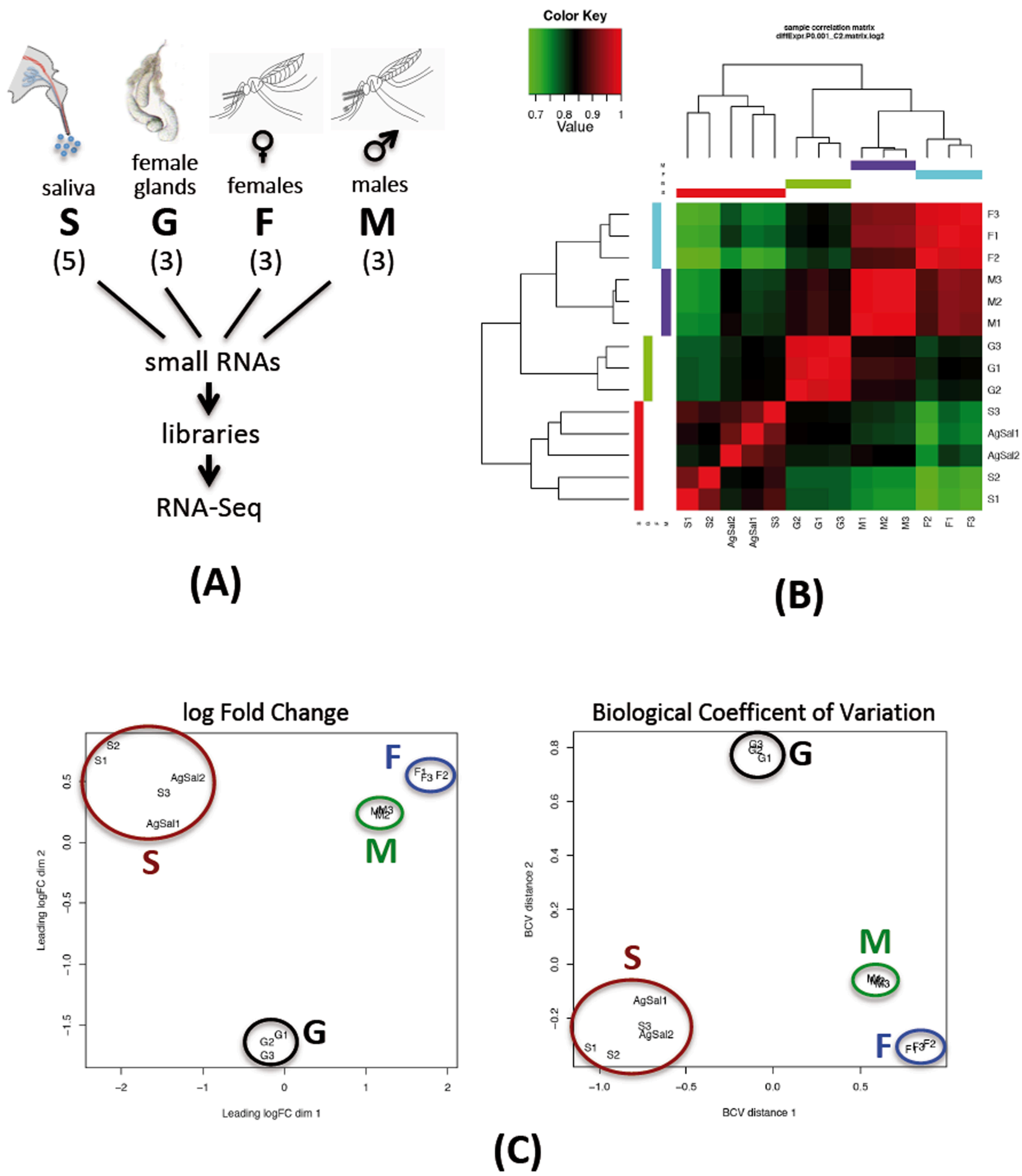

Supplementary Figure S1. Samples analyzed in this RNAseq study and correlation among replicates. (**A**) Small RNAs were extracted from saliva of adult females (S), adult female salivary glands (G), whole adult females (F) and whole adult males (M). Libraries were prepared and sequenced by Illumina small RNA-Seq. Number of biological replicates are shown in brackets. (**B**) Small RNA expression-based clustering of *An. gambiae* samples. Cells represents colour-coded Pearson correlation coefficients measuring the similarity of small RNA expression profiles between two samples. Hierarchical clustering of samples was performed based on euclidean distances between sample expression profiles. Expression values used to build the matrix were calculated as log2-transformed CPM values. Only small non-coding RNAs with CPM ≥ 1 in at least three samples were used. (**C**) Multidimensional scaling plots based on Fold Change (left) and Biological Coefficient of Variation (right). Plots were generated using the plotMDS function implemented in the edgeR software package. Only small non-coding RNAs with CPM ≥ 1 in at least three samples were used.

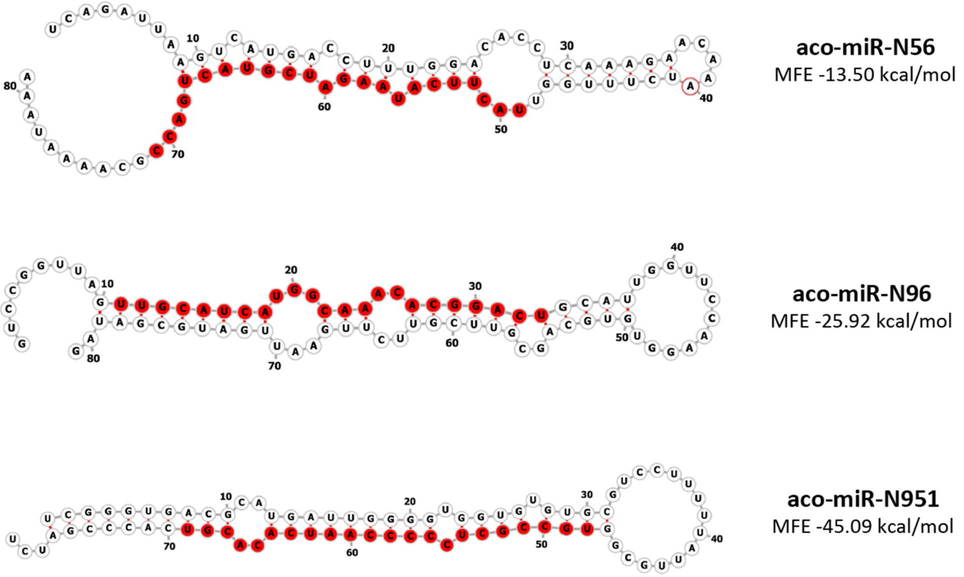

Supplemetary Figure S2. Predicted secondary structure of three novel abundant *An coluzzii* miRNA precursors. (**A**) aco-miR-N56, aco-miR-N96 and aco-miR-N951 stem-loops as predicted by RNAfold. The Minimal Free Energy (MFE) in kcal/mol is shown. The sequence of the putative mature miRNAs is highlighted in red.

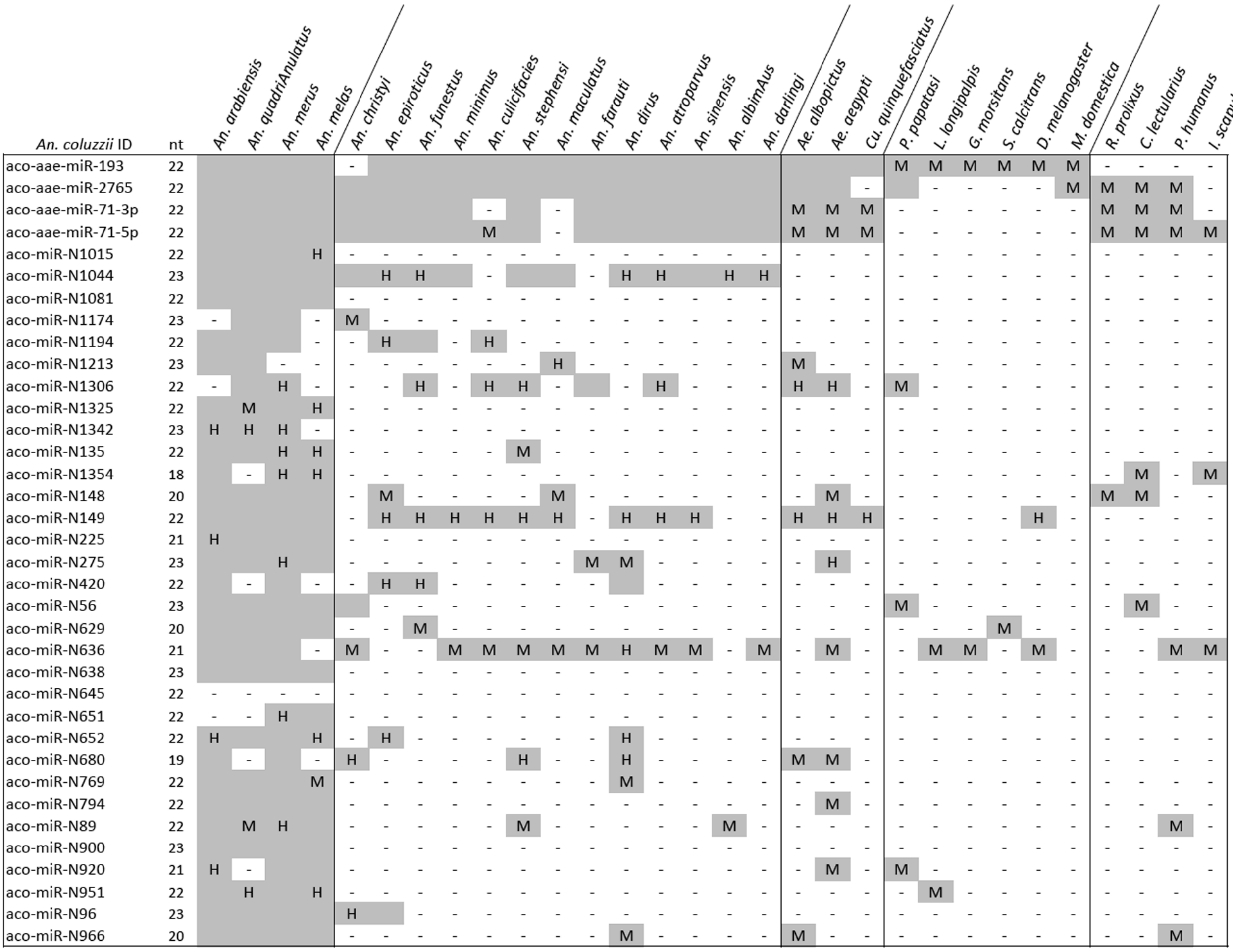

Supplementary Figure S3. Conservation of novel miRNAs in different species. Homologues of the 36 novel *An. coluzzii* miRNAs were searched in the genomes of several species by blastn using both mature and precursor miRNAs as a query. The cutoffs for inclusion were: (i) ≥70% identity over ≥70% of the length for miRNA precursors; (ii) ≥90% identity over the entire length and fully conserved seed sequence for mature miRNAs. Shaded positions indicate putative conservation: empty, both hairpin and mature conserved; H, hairpin only; M, mature only. Genomes searched included: anophelines (several species as indicated), culicines (*Aedes albopictus*, *Aedes aegypti* and *Culex quinquefasciatus*), sand flies (*Phlebotomus papatasi* and *Lutzomyia longipalpis*), the tsetse fly *Glossina morsitans*, the stable fly *Stomoxys calcitrans*, the bugs *Rhodnius prolixus* and *Cimex lectularius*, the human body louse *Pediculus humanus*, the tick *Ixodes scapularis* and the non blood feeding Diptera *Drosophila melanogaster* and *Musca domestica*.

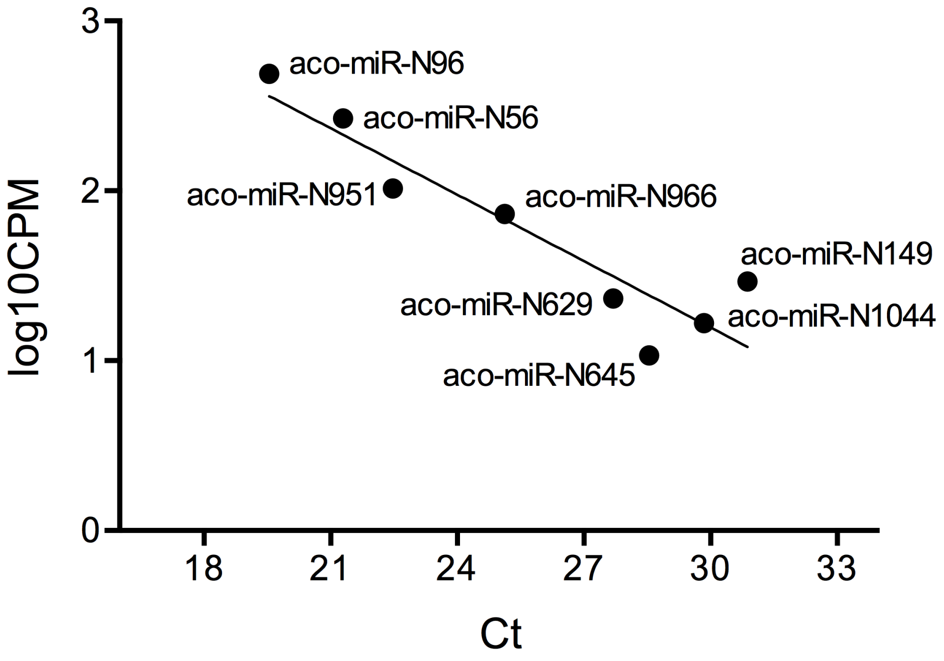

Supplemetary Figure S4. PCR validation of novel miRNA. Correlation between mean CPM as obtained by RNAseq and Ct values as determined by Stem-loop Reverse-Transcription Polymerase Chain Reaction amplification (Spearman r = -0.8333, p value = 0.0154). The linear regression line and names of the different miRNAs are shown.

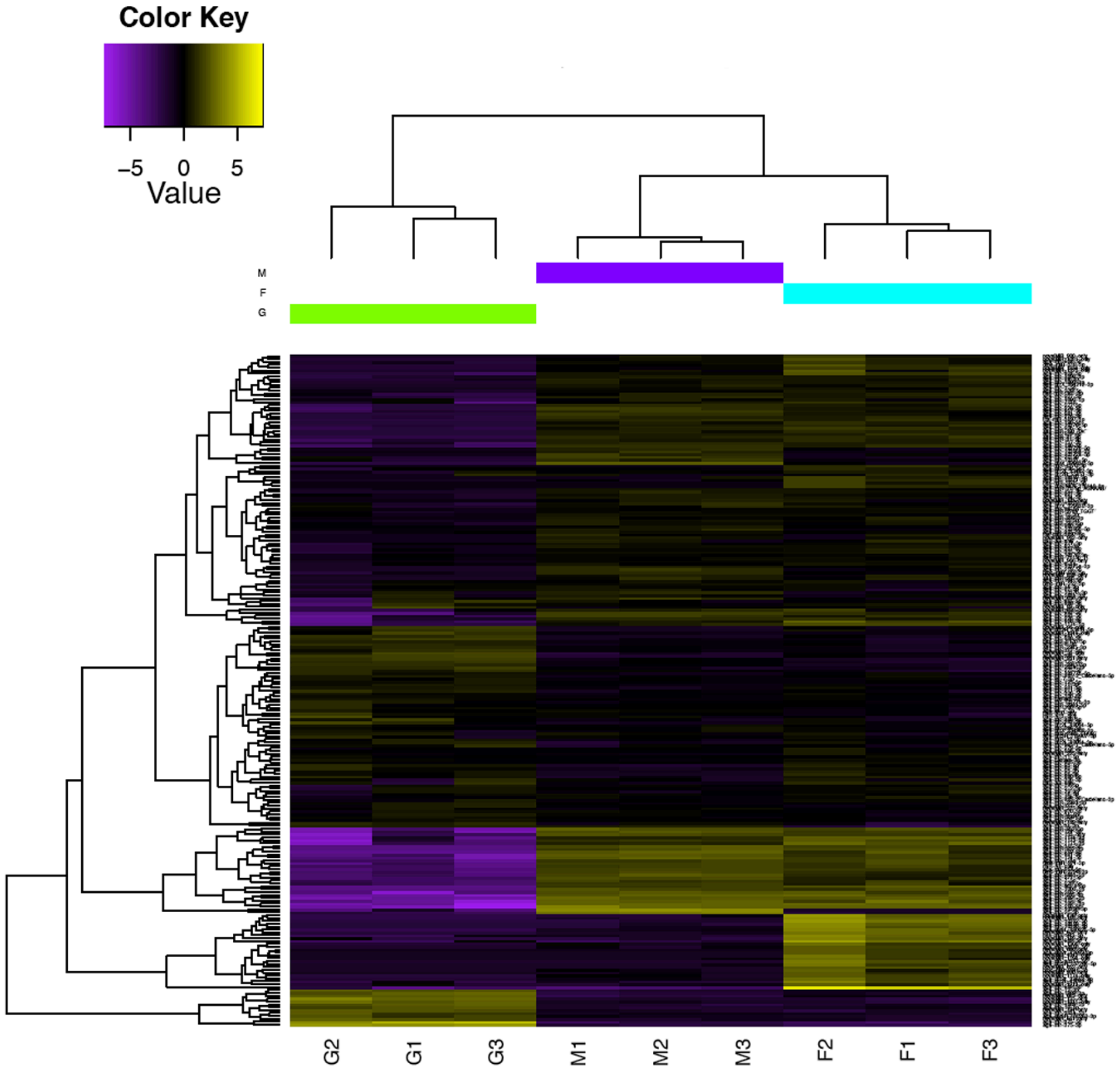

Supplemetary Figure S5. Mature miRNA expression profiles. Mature miRNA expression heatmap and hierarchical clustering of G, F and M samples. Cells correspond to mean-centered log2-transformed colour-coded CPM values. Only mature miRNAs with ≥ 1 CPM in at least three samples were used.

(A)

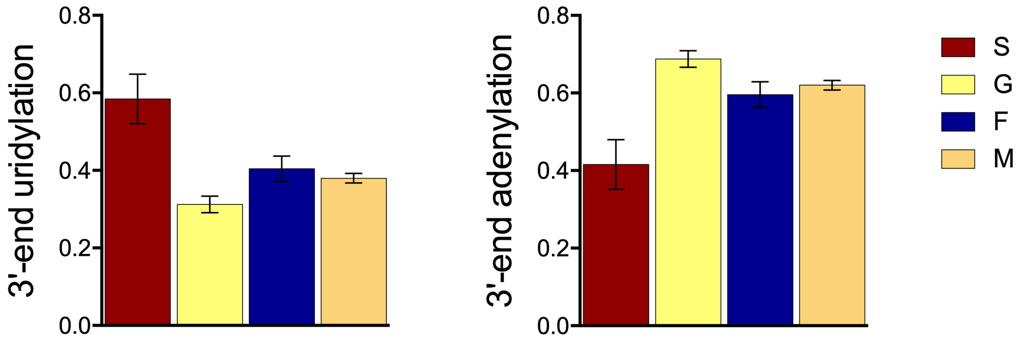

(B)

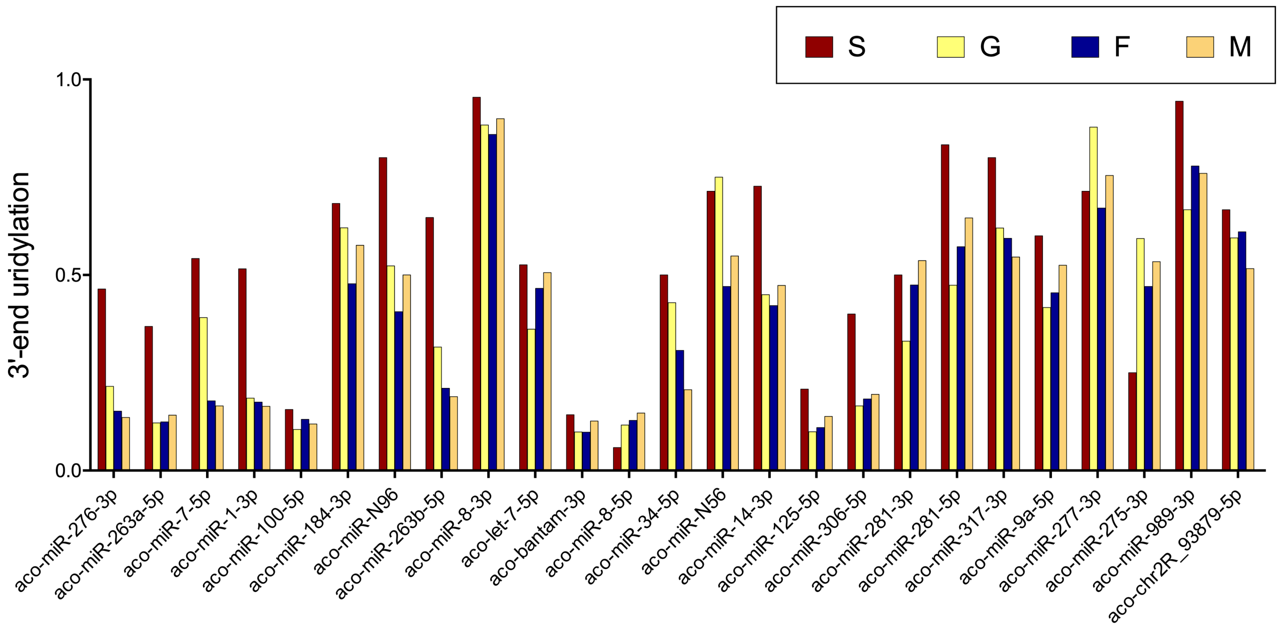

Supplementary Figure S6. Non-templated 3'-end miRNA uridylation and adenylation. The number of miRNA reads carrying non-templated 3'-end uridylation or adenylation in the saliva (S), salivary gland (G), adult female (F) and adult male (M) miRNA samples was determined. In all cases uridylation or adenylation are expressed as fraction of the total number of U+A non-templated additions. (**A**) Proportion of non-templated 3'-end uridylation (left) and adenylation (right) in the S, G, F and M samples. Mean percentages among the replicate samples are reported, with bars representing standard errors. (**B**) Fraction of uridylated miRNA reads corresponding to the 30 most abundant miRNAs from *An. coluzzii* saliva in the four samples (S, G, F and M). miRNAs are ordered from left to right according to their abundance in the saliva sample. The five miRNAs with no reads showing A or U additions in the saliva sample were not included in the figure.

Supplementary Table S1. MiRNA expression profiling by edgeR

		FDR<0.05	FDR<0.01	FDR<0.001
**G vs F**	G_{up}	38	36	33
	F_{up}	103	96	78
	tot.	160	138	111
**G vs M**	G_{up}	41	39	35
	M_{up}	84	68	60
	tot.	139	109	96
**F vs M**	F_{up}	50	48	37
	M_{up}	18	15	8
	tot.	84	69	45

Number of miRNAs upregulated (Fold Change >2) in the three different pairwise comparisons (G vs F, G vs P and F vs M) according to different False Discovery Rates (FDR <0.05, <0.01 and <0.001). Tot., total number of miRNAs below the selected FDR independently from the Fold Change.

Supplementary Table S2. Targets of human orthologues of miRNAs from *An. coluzzii* saliva.

***An. coluzzii* miRNA**	***H. sapiens* miRNA**	**target**	**PMID**
aco-miR-7-5p	hsa-miR-7-5p	EGFR	19073608
		IGF1R	25394492
		NF-kB/RelA	27203220
		RNF183	26818663
aco-miR-1-3p	hsa-miR-1-3p	MPL	24043765
		CCL2	28618950
		FN1	21924268
aco-miR-100-5p	hsa-miR-100-5p	CXCR7	27035873
		FKB51	24030073
aco-miR-184-3p	hsa-miR-184	NFAT1	19286996
		IGF1R	28927068
		FOG2	27825105
		PDGFB	27825105
aco-let-7-5p	hsa-let-7a	IL-13	24105413
		IL-6	24001203
aco-miR-92a-3p	hsa-miR-92a-3p	MKK4	23355465
		KLF2	25550450
		KLF4	25550450
		SIRT1	25550450
		CCL8	25253336
aco-miR-8-5p	hsa-miR-200b-5p	MyD88	22522429
		c-Jun	24749688
aco-miR-34-5p	hsa-miR-34c-5p	PCAF/KAT2B	27993935
aco-miR-125-5p	hsa-miR-125b-5p	IRF4	19047678
		PRDM1/BLIMP1	19047678
		TNFAIP3	22550173
		TNFα	17911593
aco-miR-10-5p	hsa-miR-10a-5p	IRAK4	25838349
		MAP3K7/TAK1	20624982
		BTRC	20624982
		IL12/IL23p40	22068236
		BCL6	22544395
aco-miR-8-3p	hsa-miR-141-3p	TRAF5	29202848
		TRAF6	29202848

The *An. coluzzi* miRNA, its human orthologue, the name of target genes and PubMed IDentifier (PMID) are reported. Whenever possible hyperlinks to the Human Protein Atlas website (www.proteinatlas.org/) and to PubMed are provided.

Supplementary Table S3. Known targets of human orthologues of miRNAs from *An. coluzzii* saliva and their involvement in immune and inflammatory responses.

***H. sapiens* miRNA**	**Targets and effects on immune and inflammatory responses**
hsa-miR-7-5p	down-regulates the NF-kB pathway (which plays a pivotal role in the production and secretion of proinflammatory cytokines) both directly, by targeting the NF-kB subunit RelA [1], and indirectly, by acting on RNF183, an ubiquitin ligase promoting the degradation of the NF-kB inhibitor IkBα [2]
hsa-miR-1-3p	targets the thrombopoietin receptor MPL, reducing the Th2- and IL-13-induced inflammatory responses [3]; also targets the inflammatory chemokine CCL2 [4], a powerful chemotactic factor involved in the recruitment of monocytes, memory T cells and natural killer (NK) cells to the sites of inflammation produced either by tissue injury or infection [5]
hsa-miR-100-5p	targets the chemokine receptor CXCR7 [6, 7]
hsa-miR-184	downregulates NFAT1, a key transcription factor that controls the expression of a wide array of cytokines (IFNγ, GM-CSF, IL-3, IL-4, IL-2, TNFα) and plays a crucial role in the initiation of Th1 immune response [8]
hsa-let-7a	regulates inflammatory and immune responses [9, 10] and may target IL-13 [11] and IL-6 [12]
hsa-miR-92a-3p	may regulate innate immunity by targeting the MAP kinase kinase 4 MKK4 [13], which is part of the TLRs signaling cascade, as well as the Kruppel-like factor 2 and 4 (KLF2, KLF4) and sirtuin 1 (SIRT1), involved in inflammasome activation and induction of endothelial innate immunity [14]; also targets CCL8 [15], a chemokine displaying chemotactic activity for monocytes, lymphocytes, basophils and eosiniphils
hsa-miR-200b-5p	targets MyD88 in macrophages, a key mediator of TLR signaling and NF-kB activation [16]
hsa-miR-34c-5p	acts as a regulator of T-cell activation [17]
hsa-miR-125b-5p	may act on interferon regulatory factor 4 (IRF4) and PRDM1, two key transcription factors in B cell differentiation [18] and also targets TNFα [19] and TNFAIP3 (TNF-alpha induced protein 3) [20], a negative regulator of NF-kB activity
hsa-miR-10a-5p	targets the p40 subunit of the heterodimeric IL-12 and IL-23 in macrophages and dendritic cells [21] as well as known regulators of NF-kB signaling as the interleukin-1 receptor-associated Kinase IRAK4 [22], the mitogen-activated protein kinase TAK1 and the beta-transducin repeat-containing gene BTRC [23]
hsa-miR-141-3p	target TNF receptor associated factor 5 and 6 (TRAF5, TRAF6) [24], which mediate TNF-induced activation of NF-kB signaling

Target genes hyperlinked to the Human Protein Atlas website (www.proteinatlas.org/).

References

1 Giles, K. M. *et al.* microRNA-7-5p inhibits melanoma cell proliferation and metastasis by suppressing RelA/NF-kappaB. *Oncotarget* **7**, 31663-31680, doi:10.18632/oncotarget.9421 (2016).

2 Yu, Q. *et al.* E3 Ubiquitin ligase RNF183 Is a Novel Regulator in Inflammatory Bowel Disease. *J Crohns Colitis* **10**, 713-725, doi:10.1093/ecco-jcc/jjw023 (2016).

3 Takyar, S. *et al.* VEGF controls lung Th2 inflammation via the miR-1-Mpl (myeloproliferative leukemia virus oncogene)-P-selectin axis. *J Exp Med* **210**, 1993-2010, doi:10.1084/jem.20121200 (2013).

4 Wang, W. *et al.* MiR-1-3p inhibits the proliferation and invasion of bladder cancer cells by suppressing CCL2 expression. *Tumour Biol* **39**, 1010428317698383, doi:10.1177/1010428317698383 (2017).

5 Deshmane, S. L., Kremlev, S., Amini, S. & Sawaya, B. E. Monocyte chemoattractant protein-1 (MCP-1): an overview. *J Interferon Cytokine Res* **29**, 313-326, doi:10.1089/jir.2008.0027 (2009).

6 Sanchez-Martin, L., Sanchez-Mateos, P. & Cabanas, C. CXCR7 impact on CXCL12 biology and disease. *Trends Mol Med* **19**, 12-22, doi:10.1016/j.molmed.2012.10.004 (2013).

7 Zhou, S. M. *et al.* miR-100 suppresses the proliferation and tumor growth of esophageal squamous cancer cells via targeting CXCR7. *Oncol Rep* **35**, 3453-3459, doi:10.3892/or.2016.4701 (2016).
8 Weitzel, R. P. *et al.* microRNA 184 regulates expression of NFAT1 in umbilical cord blood CD4+ T cells. *Blood* **113**, 6648-6657, doi:10.1182/blood-2008-09-181156 (2009).
9 Polikepahad, S. *et al.* Proinflammatory role for let-7 microRNAS in experimental asthma. *The Journal of biological chemistry* **285**, 30139-30149, doi:10.1074/jbc.M110.145698 (2010).
10 Zhang, Z. *et al.* Let-7a suppresses macrophage infiltrations and malignant phenotype of Ewing sarcoma via STAT3/NF-kappaB positive regulatory circuit. *Cancer Lett* **374**, 192-201, doi:10.1016/j.canlet.2016.02.027 (2016).
11 Jiang, L. Q. *et al.* Autocrine role of interleukin-13 on skeletal muscle glucose metabolism in type 2 diabetic patients involves microRNA let-7. *Am J Physiol Endocrinol Metab* **305**, E1359-1366, doi:10.1152/ajpendo.00236.2013 (2013).
12 Chafin, C. B., Regna, N. L., Dai, R., Caudell, D. L. & Reilly, C. M. MicroRNA-let-7a expression is increased in the mesangial cells of NZB/W mice and increases IL-6 production in vitro. *Autoimmunity* **46**, 351-362, doi:10.3109/08916934.2013.773976 (2013).
13 Lai, L. *et al.* MicroRNA-92a negatively regulates Toll-like receptor (TLR)-triggered inflammatory response in macrophages by targeting MKK4 kinase. *The Journal of biological chemistry* **288**, 7956-7967, doi:10.1074/jbc.M112.445429 (2013).
14 Chen, Z. *et al.* Oxidative stress activates endothelial innate immunity via sterol regulatory element binding protein 2 (SREBP2) transactivation of microRNA-92a. *Circulation* **131**, 805-814, doi:10.1161/CIRCULATIONAHA.114.013675 (2015).
15 Poole, E. *et al.* Latency-associated viral interleukin-10 (IL-10) encoded by human cytomegalovirus modulates cellular IL-10 and CCL8 Secretion during latent infection through changes in the cellular microRNA hsa-miR-92a. *J Virol* **88**, 13947-13955, doi:10.1128/JVI.02424-14 (2014).
16 Wendlandt, E. B., Graff, J. W., Gioannini, T. L., McCaffrey, A. P. & Wilson, M. E. The role of microRNAs miR-200b and miR-200c in TLR4 signaling and NF-kappaB activation. *Innate Immun* **18**, 846-855, doi:10.1177/1753425912443903 (2012).
17 Amaral, A. J. *et al.* miRNA profiling of human naive CD4 T cells links miR-34c-5p to cell activation and HIV replication. *EMBO J* **36**, 346-360, doi:10.15252/embj.201694335 (2017).
18 Malumbres, R. *et al.* Differentiation stage-specific expression of microRNAs in B lymphocytes and diffuse large B-cell lymphomas. *Blood* **113**, 3754-3764, doi:10.1182/blood-2008-10-184077 (2009).
19 Tili, E. *et al.* Modulation of miR-155 and miR-125b levels following lipopolysaccharide/TNF-alpha stimulation and their possible roles in regulating the response to endotoxin shock. *J Immunol* **179**, 5082-5089 (2007).
20 Kim, S. W. *et al.* MicroRNAs miR-125a and miR-125b constitutively activate the NF-kappaB pathway by targeting the tumor necrosis factor alpha-induced protein 3 (TNFAIP3, A20). *Proc Natl Acad Sci U S A* **109**, 7865-7870, doi:10.1073/pnas.1200081109 (2012).
21 Xue, X. *et al.* Microbiota downregulates dendritic cell expression of miR-10a, which targets IL-12/IL-23p40. *J Immunol* **187**, 5879-5886, doi:10.4049/jimmunol.1100535 (2011).
22 Njock, M. S. *et al.* Endothelial cells suppress monocyte activation through secretion of extracellular vesicles containing antiinflammatory microRNAs. *Blood* **125**, 3202-3212, doi:10.1182/blood-2014-11-611046 (2015).
23 Fang, Y., Shi, C., Manduchi, E., Civelek, M. & Davies, P. F. MicroRNA-10a regulation of proinflammatory phenotype in athero-susceptible endothelium in vivo and in vitro. *Proc Natl Acad Sci U S A* **107**, 13450-13455, doi:10.1073/pnas.1002120107 (2010).
24 Huang, S. *et al.* Downregulation of miR-141-3p promotes bone metastasis via activating NF-kappaB signaling in prostate cancer. *J Exp Clin Cancer Res* **36**, 173, doi:10.1186/s13046-017-0645-7 (2017).

Supplementary Table S4. Differentially abundant *An. coluzzii* miRNAs.

G_{enr} (G vs F)	G_{enr} (G vs M)	M_{enr} (M vs F)	F_{enr} (M vs F)	G_{enr} (G vs F) and S
**aco-chr3L_118320-5p**	aco-chr2L_48064-3p	aco-miR-10368-3p	aco-miR-283-5p	aco-chr3R_160384-3p
**aco-chr3R_160262-3p**	aco-chr2R_55451-3p	aco-miR-2c-3p	aco-miR-9c-5p	aco-miR-100-5p
**aco-chr3R_160384-3p**	**aco-chr3L_118320-5p**	aco-miR-10366b-5p	aco-miR-N56	aco-miR-125-5p
**aco-chr3R_160384-5p**	**aco-chr3R_160262-3p**	aco-chrX_329630-5p	aco-miR-1174-5p	aco-miR-263a-5p
aco-chrX_353716-3p	**aco-chr3R_160384-3p**	aco-miR-10366a-3p	aco-chr2R_55451-3p	aco-miR-281-5p
aco-miR-100-5p	**aco-chr3R_160384-5p**	aco-miR-2c-5p	aco-miR-13b-5p	aco-miR-317-3p
**aco-miR-10377b-3p**	aco-chrX_353198-3p	aco-miR-10372a-5p	aco-miR-N225	aco-miR-317-5p
**aco-miR-12-3p**	**aco-miR-10377b-3p**	aco-miR-10370-3p	aco-chr2L_48064-3p	aco-miR-8-5p
**aco-miR-12-5p**	aco-miR-11-3p	aco-miR-34-3p	aco-miR-980-5p	aco-miR-N56
**aco-miR-125-5p**	**aco-miR-12-3p**	aco-miR-10372b-5p	aco-miR-92a-3p	aco-miR-N96
**aco-miR-1889-3p**	**aco-miR-12-5p**	aco-miR-10367-5p	aco-miR-1174-3p	
**aco-miR-1889-5p**	**aco-miR-125-5p**	aco-miR-N636	aco-miR-N645	
**aco-miR-2-1-5p**	**aco-miR-1889-3p**	aco-miR-219-5p	aco-miR-1175-3p	
**aco-miR-263a-3p**	**aco-miR-1889-5p**	aco-miR-263b-5p	aco-miR-9c-3p	
**aco-miR-263a-5p**	**aco-miR-2-1-5p**	aco-chr2R_93879-5p	aco-miR-92b-3p	
aco-miR-275-5p	**aco-miR-263a-3p**	aco-miR-275-5p	aco-miR-9b-5p	
**aco-miR-281-5p**	**aco-miR-263a-5p**	aco-miR-981-5p	aco-chr2L_1641-3p	
**aco-miR-283-3p**	aco-miR-281-3p	aco-miR-10379-3p	aco-miR-N1354	
**aco-miR-283-5p**	**aco-miR-281-5p**		aco-miR-10359-3p	
**aco-miR-317-3p**	**aco-miR-283-3p**		aco-miR-9b-3p	
aco-miR-317-5p	**aco-miR-283-5p**		aco-miR-10371-5p	
aco-miR-34-3p	aco-miR-308-3p		aco-miR-N1174	
**aco-miR-375-3p**	**aco-miR-317-3p**		aco-miR-N1306	
**aco-miR-375-5p**	**aco-miR-375-3p**		aco-chr2L_4162-5p	
**aco-miR-8-5p**	**aco-miR-375-5p**		aco-miR-10358-5p	
**aco-miR-988-3p**	**aco-miR-8-5p**		aco-chr2L_32652-3p	
aco-miR-N10	aco-miR-965-2-5p		aco-miR-N1213	
**aco-miR-N1015**	aco-miR-980-5p		aco-chr3L_130625-5p	
**aco-miR-N1044**	**aco-miR-988-3p**		aco-chrUNKN_283499-3p	
aco-miR-N13	aco-miR-9b-5p		aco-miR-N920	
**aco-miR-N135**	aco-miR-9c-3p		aco-miR-N1194	
**aco-miR-N56**	aco-miR-9c-5p		aco-chr3R_150582-5p	
**aco-miR-N645**	**aco-miR-N1015**		aco-miR-N629	
**aco-miR-N651**	**aco-miR-N1044**		aco-chrUNKN_316148-3p	
aco-miR-N769	**aco-miR-N135**		aco-chrUNKN_283499-5p	
**aco-miR-N794**	**aco-miR-N56**		aco-miR-10360-5p	
**aco-miR-N951**	**aco-miR-N645**		aco-chr2L_42099-5p	
**aco-miR-N96**	**aco-miR-N651**		aco-miR-N420	
	**aco-miR-N794**		aco-chr3R_157264-5p	
	**aco-miR-N951**		aco-miR-N148	
	**aco-miR-N96**		aco-miR-N680	
			aco-chr2L_42099-3p	
			aco-miR-N1342	
			aco-miR-N652	
			aco-miR-N149	
			aco-miR-10359-5p	
			aco-miR-989-5p	
			aco-miR-10362-5p	
			aco-miR-10357-5p	
			aco-miR-989-3p	

Column 1 and 2: miRNAs G-enriched in the pairwise comparisons G-F and G-M (common miRNAs in bold). Columns 3 and 4: miRNAs M-enriched (column 3) and F-enriched (column 4) in the comparisons M-F. Column 5: miRNAs G-enriched in the G-F comparison and abundant in saliva (among top 30). miRNAs were considered enriched when FC>2 and FDR<0.05.

Supplementary Table S5. List of primers used for reverse transcription and qPCR.

miRNA	Primer	Sequence
aco-miR-N96	SLRT-96<br>F-96	5'-GTCGTATCCAGTGCAGGGTCCGAGGTATTCGCACTGGATACGACAGTCCG-3'OH<br>5'-CACGCATTGCATCATGGCAAACA-3'OH
aco-miR-N56	SLRT-56<br>F-56	5'-GTCGTATCCAGTGCAGGGTCCGAGGTATTCGCACTGGATACGACGGTCAG-3'OH<br>5'-GTCCACGCATACTTCATAAGATCGTA-3'OH
aco-miR-N951	SLRT-951<br>F-951	5'-GTCGTATCCAGTGCAGGGTCCGAGGTATTCGCACTGGATACGACACGTGT-3'OH<br>5'-CACGCATGCCGCTCCCCCAATC-3'OH
aco-miR-N966	SLRT-966<br>F-966	5'-GTCGTATCCAGTGCAGGGTCCGAGGTATTCGCACTGGATACGACTTCAGC-3'OH<br>5'-CAGTCCACGCATGAGTCGTTATCAT-3'OH
aco-miR-N135	SLRT-135<br>F-135	5'-GTCGTATCCAGTGCAGGGTCCGAGGTATTCGCACTGGATACGACTCCGAA-3'OH<br>5'-CACGCATCGGACACACACACCC-3'OH
aco-miR-N1044	SLRT-1044<br>F-1044	5'-GTCGTATCCAGTGCAGGGTCCGAGGTATTCGCACTGGATACGACAAATGT-3'OH<br>5'-GTCCACGCAAAGCTACACCTGGTTGA-3'OH
aco-miR-N645	SLRT-645<br>F-645	5'-GTCGTATCCAGTGCAGGGTCCGAGGTATTCGCACTGGATACGACCTCGGA-3'OH<br>5'-GTCCACGCATAGAGCCGTCTAACTT-3'OH
aco-miR-N149	SLRT-149<br>F-149	5'-GTCGTATCCAGTGCAGGGTCCGAGGTATTCGCACTGGATACGACGCTCCC-3'OH<br>5'-AGCGTCCACGCATAATGGAAATTTATCA-3'OH
aco-miR-N629	SLRT-629<br>F-629	5'-GTCGTATCCAGTGCAGGGTCCGAGGTATTCGCACTGGATACGACGCTACA-3'OH<br>5'-GTCCACGCACAAGAGTGTCCAGC-3'OH
Universal reverse	Uni_Rev	5'-CCAGTGCAGGGTCCGAGGTA-3'OH

Legends to Supplementary Files

Supplementary file S1. List of hairpins and mature miRNAs used for mapping. The worksheet aga_hairpins includes a total of 273 hairpins: 175 previously known *An. gambiae* miRNA precursors (66 from miRBase, 59 from Biryukova I. *et al.* [53], 41 from Castellano L. *et al.* [54], 9 from Fu X. *et al.* [55]) plus 39 predicted by miRDeep* [69] and 59 predicted by MapMi [70 70]. The worksheet aga_mature includes 131 *An. gambiae* miRNAs from miRBase, 118 from Biryukova I. *et al.* [53], 81 from Castellano L. *et al.* [54], 9 from Fu *et al.* [55], 39 predicted by miRDeep* [69] and 60 predicted by MapMi [70]. ID indicates the name of the miRNA according to miRBase, to previous studies or to mature miRNAs used as input for the MapMi program. Source, miRBase accession (when available), genomic coordinates and sequences are also provided.

Supplementary file S2. Lists of expressed mature *An. coluzzii* miRNAs. The six worksheets include: the complete list of 214 miRNAs (aco_mature_214); the lists of miRNAs found in saliva (aco_S_77), in salivary glands (aco_G_147), in adult females (aco_F_196) and in adult males (aco_M_171); the lists of miRNAs found exclusively in G, F and M (G_F_M_only). Only miRNAs with counts in at least 2 of the 3 replicates (3 of 5 for the S sample) and a mean CPM ≥ 3 in at least one of the four samples are included. For each miRNA the *An. coluzzii* ID, the *An. gambiae* ID, the source, the miRBase and VectorBase accession numbers (when available), the sequence and length of the mature miRNA as well as counts and CPM are reported.

Supplementary file S3. List of the 36 putative novel *Anopheles coluzzi* miRNAs. The *An. coluzzii* ID, the source (miRDeep* or MapMi), VectorBase accession numbers (if available), the sequence of mature miRNAs, arm, length, the total counts in the 14 libraries, the number of libraries, counts and CPM in the different libraries, the sequence of their precursors with the minimal free energy (MFE) of the predicted secondary structures are reported.

Supplementary file S4. Differential Expression analysis. For each pairwise comparison (G-F, G-M, M-F) differential expression data as obtained by edgeR and with three different cut off (FDR <0.05, FDR <0.01 and FDR <0.001) are reported.

Supplementary file S5. Combined list of the 30 most abundant miRNAs in saliva and salivary glands. The list was compiled comparing the 30 most abundant miRNAs in saliva and salivary glands of *An. coluzzii*. For the resulting 39 miRNAs the mean CPM in saliva and salivary glands, the S/G ratio and the results of differential expression analysis by edgeR are shown. The 21 miRNAs common to both lists are highlighted (grey shading). miRNAs with S/G ratio > 4.0 and < 0.25 are shaded in red and light blue, respectively. The remaining miRNAs (0.25 ≤ S/G ratio ≤ 4.0) are shaded in green. Source, miRBase and VectorBase accession, sequence, length, counts and CPM in individual replicates of all samples are also included (columns hided for clarity).

Supplementary file S6. Conservation of the top 30 *Anopheles coluzzii* saliva miRNAs in saliva or exosomes from other species. The presence of the top 30 *An. coluzzi* saliva miRNAs in the saliva of humans, of the mosquitoes *Ae. aegypti* and *Ae. albopictus*, of the tick *I. ricinus* and in exosomal vesicles secreted by the parasitic nematodes *B. malayi* and *H. polygyrus* is reported. miRNAs were considered as present if among the top 50 in human saliva or the top 30 in all other cases. Presence in saliva or in exosomes, seed conservation, number of mismatches (mm) and rank are shown. *An. coluzzi* miRNAs mimicking human miRNAs and their orthologues in saliva or exosomes of the different species are highlighted in grey. Positions within the first 50 (human) or 30 (other) are highlighted in green.
